# MKLN1-AS promotes pancreatic cancer progression as a crucial downstream mediator of HIF-1α through miR-185-5p/TEAD1 pathway

**DOI:** 10.1007/s10565-024-09863-8

**Published:** 2024-05-13

**Authors:** Jiayu Chen, Lei Li, Yongpu Feng, Yating Zhao, Fengyuan Sun, Xianzhu Zhou, Du Yiqi, Zhaoshen Li, Fanyang Kong, Xiangyu Kong

**Affiliations:** 1 Department of Gastroenterology, Changhai Hospital, Naval Medical University, Shanghai, 200433 China; 2 Shanghai Institute of Pancreatic Diseases, Shanghai, 200433 China; 3 National key laboratory of Immunity and inflammation, Naval Medical University, Shanghai, 200433 China; 4 Digestive Endoscopy Center, Shanghai Tenth People’s Hospital, Tongji University School of Medicine, Shanghai, 200433 China

**Keywords:** PDAC, hypoxia, lncRNA, MKLN1-AS, TEAD1

## Abstract

In pancreatic ductal adenocarcinomas (PDAC), profound hypoxia plays key roles in regulating cancer cell behavior, including proliferation, migration, and resistance to therapies. The initial part of this research highlights the important role played by long noncoding RNA (lncRNA) MKLN1-AS, which is controlled by hypoxia-inducible factor-1 alpha (HIF-1α), in the progression of PDAC. Human samples of PDAC showed a notable increase in MKLN1-AS expression, which was linked to a worse outcome. Forced expression of MKLN1-AS greatly reduced the inhibitory impact on the growth and spread of PDAC cells caused by HIF-1α depletion. Experiments on mechanisms showed that HIF-1α influences the expression of MKLN1-AS by directly attaching to a hypoxia response element in the promoter region of MKLN1-AS.MKLN1-AS acts as a competitive endogenous RNA (ceRNA) by binding to miR-185-5p, resulting in the regulation of TEAD1 expression and promoting cell proliferation, migration, and tumor growth. TEAD1 subsequently enhances the development of PDAC. Our study results suggest that MKLN1-AS could serve as a promising target for treatment and a valuable indicator for predicting outcomes in PDAC. PDAC is associated with low oxygen levels, and the long non-coding RNA MKLN1-AS interacts with TEAD1 in this context.

**Graphical abstract:**

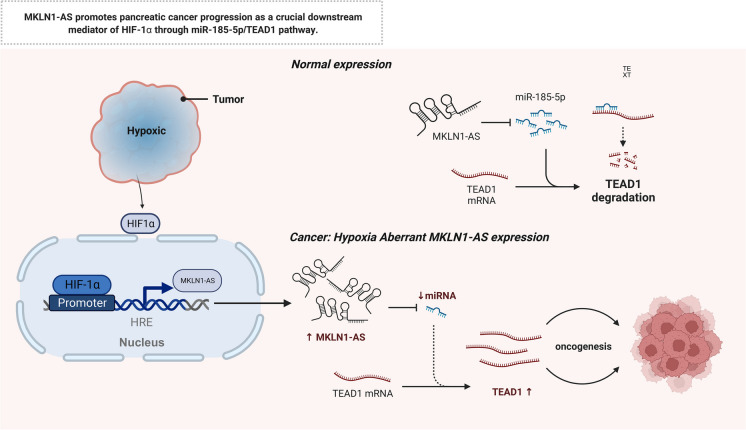

**Supplementary Information:**

The online version contains supplementary material available at 10.1007/s10565-024-09863-8.

## Introduction

Pancreatic cancer, holding the seventh position in cancer-related deaths, stands as one of the deadliest human cancers (Siegel et al. 2022). Pancreatic ductal adenocarcinoma (PDAC) is the most common type of pancreatic cancer, representing 90% of cases (Park et al. 2021). The poor prognosis of PDAC has been attributed to a variety of factors, including challenges in early detection and resistance to drugs (Connor and Gallinger 2022; Cai et al. 2021). Currently, available effective therapeutics against PDAC remain limited. Thus, the investigation of targeted molecular therapies is urgently needed to improve outcomes of PDAC.

The hypoxic tumor microenvironment is a prevalent characteristic of PDAC, that contributes to tumor progression and negatively impacts on prognosis (Tao et al. 2021). Uncontrolled tumor proliferation and fibrosis formation result in intratumoral necrosis and insufficient oxygen supply in PDAC (Park et al. 2021). Oxygen availability greatly influences the expression and activity of HIF-1α. Unlike normoxic environments, PHD-containing proteins quickly hydroxylate HIF-1α, causing it to be polyubiquitinated by the tumor suppressor pVHL (Semenza 2001). This results in its accumulation in the nucleus during hypoxic conditions, leading to nuclear translocation. Nuclear HIF-1α forms a heterodimer with HIF-β, facilitating the binding to hypoxia-responsive elements (HREs) in the promoters and thereby promoting the transcription of hypoxia-responsive genes (Kong et al. 2017).

Long non-coding RNAs (lncRNAs) are RNA molecules that consist of more than 200 nucleotides. Multiple studies have shown that the abnormal expression of lncRNAs plays a role in various biological functions in cancer cells, such as growth, movement, metabolic processes, cell death, and resistance to drugs (Tan et al. 2021; Liu et al. 2020; Zhao et al. 2020; Chen et al. 2021). Hypoxia-induced activation of HIF-1α mediated mounting aspects of oncogenic mechanisms to regulate cancer development, especially lncRNA dysregulation. Hypoxia-responsive lncRNAs are transcribed under the regulation of HIF-1α. Previous studies have reported activation of lncRNAs STEAP3-AS1 and LUCAT1 by HIF-1α leads to promoted tumor progression (Huan et al. 2020; Zhou et al. 2022). However, therapeutic strategies targeting HIF-1α signaling have shown limited efficacy (Lee et al. 2016), targeting HIF-1α downstream effector molecules can be a potentially successful alternative approach in PDAC treatment.

The present research shows that a hypoxia-inducible lncRNA named MKLN1-AS is upregulated in PDAC tumor tissues. Increased expression of MKLN1-AS was associated with unfavorable outcomes in individuals with PDAC, aligning with earlier research indicating the involvement of MKLN1-AS in the advancement of hepatocellular carcinoma (Gao et al. 2020). Our study aims to elucidate the biological functions of MKLN1-AS in PDAC, which is governed by the regulation of HIF-1α. Our findings indicated that elevated levels of MKLN1-AS in PDAC act as a competitive endogenous RNA (ceRNA) by interacting with miR-185-5p, resulting in enhanced cell proliferation, migration, and tumor development through the modulation of TEAD1 expression.

## Materials and methods

### Patients and tissue species

Twenty-five people diagnosed with PDAC at Changhai Hospital provided tumor tissues and adjacent non-cancerous pancreatic tissues for analysis. The participants in the study were granted written informed consent. The features of the patients were recorded in Supplementary Table 1. The diagnosis of PDAC in all patients was conclusively confirmed through surgical procedures and pathological examinations. Before the surgical procedure, none of the patients had received radiotherapy or chemotherapy treatments. Upon receiving the tissue samples from patients, they were promptly frozen in liquid nitrogen and stored at -80°C.

### Extraction of RNA and performing real-time PCR

RNA was extracted from tissue and cell cultures with TRIzol® reagent (Invitrogen, MA, USA). RNA concentrations and quality were assessed using UV spectrophotometry. RNAs were reverse transcribed using the HiScript® III 1st Strand cDNA Synthesis Kit from Vazyme in Nanjing, China, following the provided guidelines. The quantitative real-time PCR (qPCR) assay was conducted using the SYBR-green mastermix (Vazyme, Nanjing, China) and done on LightCycler 480II equipment (Roche, Mannheim, Germany).In both mRNA and lncRNA, endogenous control was utilized via b-actin. The qPCR temperature procedure includes starting with a denaturation step at 95°C for 5 minutes, then proceeding with 40 cycles of temperature fluctuation between 95°C and 65°C for 45 seconds each.cDNA synthesis and qPCR were used to assess the expression levels of microR-185-5p and microR-148b-3p, with the miScript RT kit (Qiagen GmbH) and miScript SYBR Green PCR kit (Qiagen GmbH) being employed, respectively. PCR primers can be found in Supplementary Table 4.

### Cell culture and treatment

Cell lines from the American Type Culture Collection, including HPNE, PANC-1, AsPC-1, CaPAN-2, SW1990, BxPC-3, and MIA PaCa-2, were acquired for the study. Cells were maintained in Dulbecco's modified Eagle's medium (DMEM) with 10% fetal bovine serum (Invitrogen, Carlsbad, CA, USA) and 1% penicillin/streptomycin. Cells were cultured at 37 °C in the atmosphere with 5% carbon dioxide.

In the hypoxic culture experiment, the cells were subjected to an incubation environment consisting of 94% nitrogen (N_2_), 1% oxygen (O_2_), and 5% carbon dioxide (CO_2_). The treatment of 250μM cobalt chloride (CoCl_2_) for 24 hours induced hypoxia.

Subcellular RNA extraction was performed according to the manufacturer's instructions using a PARISTM Kit (Ambion). Quantitative reverse transcription polymerase chain reaction (qRT-PCR) was used to further analyze RNA in the cytoplasm and nucleus. β-actin and U6 served as controls for cytoplasmic and nuclear compartments, respectively.

### Oligonucleotides and transfection

In Shanghai, China, GenePharma created vectors for overexpression, shRNA, and siRNA that target HIF-1α, MKLN1-AS, and TEAD1. A lentivirus vector carrying these sequences (GenePharma, Shanghai, China) was also constructed containing matching controls. MiR-185-5p mimics, miR-185-5p inhibitor, and their respective negative controls were supplied by GenePharma in Shanghai, China. PDAC cells were transfected with the oligonucleotides using Lipofectamine 3000 (Invitrogen, USA), followed by confirmation of transfection efficiency through qRT-PCR analysis.

### Western blot analysis

The cell lysate was created by treating the cells with RIPA buffer from Epizyme in Shanghai, China, and the amount of protein was measured using the BCA Protein Quantification Kit. A 30 μg protein sample was subjected to electrophoretic separation on a 12% SDS-polyacrylamide gel. After electrophoresis, the proteins were moved to PVDF membranes. Following incubation with a 5% skim milk solution in TBS-T for four hours at room temperature, primary antibodies against TEAD1 (diluted 1:2000, purchased from Abcam, catalog number ab133533, UK) and HIF-1α (diluted 1:2000, acquired from Abcam, catalog number ab79546, UK) were left overnight at 4°C. To ensure equal loading of protein samples, an anti-β-actin antibody (diluted at 1:5000, sourced from Abcam, catalog number ab6275, United Kingdom) was employed. After washing the membranes three times with TBS-T for 15 minutes each, they were then incubated with secondary antibodies (diluted at 1:10,000; Westang) conjugated to horseradish peroxidase for 45 minutes at room temperature. Protein bands were visualized using a developing solution (Vazyme, Nanjing, China) and exposed to X-ray film.

### Bioinformatic analysis

Data on clinical characteristics and gene expression in relation to PDAC were acquired from The Cancer Genome Atlas (TCGA) database and Gene Expression Omnibus (GEO) database, available at https://portal.gdc.cancer.gov/ and https://www.ncbi.nlm.nih.gov/geo/ respectively. All data included 171 PDAC patients from the TCGA database and 63 PDAC patients from the GEO database, and the quality controls were analyzed by R language. The details of data from the public database are exhibited in Supplementary Tables 2, and 3. Levels of gene expression were standardized and then divided into groups based on a predetermined threshold to distinguish between high and low expression. Correlation maps involving two genes were generated using the ggstatsplot software in R, while heatmaps displaying correlations among multiple genes were visualized using the R software package. Molecular interactions and relevant clinical data were analyzed using the R programming language.

Shared microRNAs (miRNAs) between MKLN1-AS and TEAD1 were identified using the miRcode and TargetScan databases, available at http://www.mircode.org/index.php and http://www.targetscan.org/vert_72/, respectively. The downstream miRNA and binding site sequences of MKLN1-AS were obtained from the JEFFERSON database, accessible at https://cm.jefferson.edu/rna22/Interactive/.

### Cell counting kit-8 (CCK-8) assay

Cell counting kit-8 (CCK-8) assay was performed to count viable cells. A controlled climate incubation environment at 37°C for 2 hours was used for incubation of cells in 96-well microtiter plates. Cells were placed in 96-well microtiter plates at a concentration of 2×10^3^ cells per well and kept at 37°C for 2 hours in a regulated incubation setting. After a 24-hour incubation period, every well was treated with 10 microliters of the Cell Counting Kit-8 (CCK-8, Dojindo, Japan). Following incubation, the plates were washed twice with phosphate-buffered saline (PBS). Afterward, absorbance at 450 nm was measured using a microplate reader following the guidelines provided by the manufacturer.

### Colony formation assay

Pancreatic cancer cells were placed in 6-well dishes with a concentration of 1×10^3^ cells per well. Colonies with a cell count exceeding 200 cells per colony were subjected to a 10-day incubation period, followed by fixation for 15 minutes using a 4% paraformaldehyde solution. Afterward, the colonies were treated with a 0.1% crystal violet solution for a duration of 15 minutes. The number of colonies in each well was quantified using Image J analysis software (version 1.48), and the mean colony area (mm^2^) was computed to facilitate comparisons of size. Three replicates of each experiment were conducted.

### EdU assay

The experiment involving EdU (5-ethynyl-2'-deoxyuridine) proliferation was carried out following the guidelines provided by the manufacturer, utilizing the Cell-Light EdU Apollo 567 In Vitro Imaging Kit from Ribobio. Briefly, cells were processed according to the suggested procedures and then placed in 96-well plates at a density of around 5×10^3^ cells per well. Each well received 100 microliters of medium containing 50 micromolar (μM) EdU, 24 hours after seeding. Afterward, the cells were placed in an incubator at a temperature of 37 degrees Celsius for a duration of 2 hours. After the cells were incubated, they were treated with a 4% paraformaldehyde solution and then stained with a mixture of Hoechst and Apollo reagents. Images were acquired using fluorescence microscopy and subsequently merged with Image J analysis software (version 1.48). Relative Edu incorporation was used for quantification analysis, followed by determining the total cell count and the count of EdU-positive cells in each field.

### Cell migration/invasion assay

The test was performed in Boyden chambers with 24 wells, each holding 12 Millipore inserts. These inserts had an 8 mm pore size and were coated with a layer of basement membrane matrix on top, or left uncoated. Tests were performed in Boyden chambers using 24-well tissue culture plates, each equipped with 12 Millipore inserts. These inserts had a polycarbonate membrane with 8μm pores and were coated with either an ECMatrix layer (for invasion experiments) or left uncoated (for migration experiments). The bottom compartments were filled with a solution that included 10% fetal bovine serum, which acted as a chemoattractant.300 μL of serum-free medium contained 5 × 105 cells in the chambers above. Cells that infiltrated the ECMatrix or moved through the polycarbonate membrane were observed and documented under a microscope following 48 hours of incubation at 37°C.

### Scratch wound healing assays

Pancreatic cancer cells were grown in 6-well dishes until they covered the surface, then manually scraped using a 20μl pipette tip. Next, a wound was induced by the addition of phosphate-buffered saline (PBS). A wound was created by manually scratching a cell monolayer with a 20-μl pipette tip, then rinsing with phosphate-buffered saline (PBS). Afterward, the cells were cultured for at least 12 hours in a medium containing 1% FBS, a level that does not impact the growth of pancreatic cancer cells. We established specific time points for capturing images within the cell growth cycle. Images of the wound healing process were captured using a phase contrast microscope. Image J analysis software (version 1.48) was utilized to measure the regions where cells migrated.

### In vivo assay

Male BALB/C athymic nude mice, five weeks old, were maintained in an environment devoid of pathogens. The Animal Research Ethics Committee at Second Military Medical University approved the animal study in advance. To monitor tumor growth progression, we administered 0.1 ml of Hank's balanced salt solution into the flank of every mouse, utilizing 1 × 10^6^ MIA PaCa-2 and SW1990 cells. Tumor volumes were measured weekly using the formula length × width^2^ × 0.5. Following a period of four weeks, the mice were put to death, and the tumors were removed.An extra set of mice received injections of MIA PaCa-2 and SW1990 cells (1× 10^6^ each) via their ileocolic veins four weeks later for the metastasis test. All mice were euthanized on the 28th-day post-injection or upon reaching a pre-mortal state. Liver resection was performed, and the number of surface metastatic lesions on the livers was quantified.

### Construction of MKLN1-AS promoter reporter plasmids

The MKLN1-AS segment, spanning from -891 to -4 in relation to the start of transcription, was inserted into a pGL3-basic vector (Promega), resulting in a 1000-bp DNA piece. This facilitated the generation of the reporter plasmid, designated as pGL3-891, which was purposefully engineered to encompass multiple hypoxia response elements (HREs). The nomenclature for the promoter-reporter plasmids was systematically based on the specific initiation site of the HREs. Subsequently, deletion mutation reporters were meticulously crafted for the plasmids, namely pGL3-681 and pGL3-30.To validate the integrity of these constructs, comprehensive sequencing was conducted on both the inserted sequences and the flanking regions of the plasmids.

### RNA immunoprecipitation (RIP)

The RIP method was carried out using the Magna RIP™ RNA-Binding Protein Immunoprecipitation Kit from Millipore in Germany, following the instructions given, and abiding by the guidelines provided by the producer. Roughly 10 million SW1990 and MIA PaCa-2 cells were lysed using RIP lysis buffer. Following centrifugation at 10,000 times gravity and 4 degrees Celsius for 5 minutes, the PDAC cells were exposed to magnetic beads linked with human Ago2 antibodies obtained from Abcam in Cambridge, UK, or control IgG antibodies from Millipore in the USA. This overnight incubation occurred at 4°C. Subsequently, the samples were treated with Proteinase K while agitating to facilitate protein digestion. The immunoprecipitated RNAs were then extracted and subjected to qRT-PCR analysis.

### Chromatin immunoprecipitation assay

The chromatin immunoprecipitation assay kit (Millipore) was used to prepare 2×10^6^ PDAC cells as specified in the manufacturer's instructions for the chromatin immunoprecipitation (ChIP) assay. DNA samples were first treated to induce precipitation before undergoing PCR amplification aimed at specific regions of the MKLN1-AS promoter. The resulting PCR products were then separated by electrophoresis on a 2% agarose gel and visualized with ethidium bromide staining.

### Promoter construction and Dual-luciferase reporter assay

The promoter sequence of MKLN1-AS was identified using the UCSC gene browser, accessible at https://genome.ucsc.edu/. To determine the binding site sequence responsible for interacting with a transcription factor and facilitating MKLN1-AS transcription, a search was conducted on the JASPAR database (https://jaspar.genereg.net). Chip-seq data for transcription factors were acquired from Cistrome Data Browser at http://cistrome.org/db.

Recombinant luciferase plasmids were created by inserting the target fragments of MKLN1-AS or TEAD1 3’-UTR sequence, with wild-type (WT) or mutant-type (MT) miR-185-5p binding sites, into the pGL3-Basic luciferase vector from Promega in the USA. Transfection of cells was performed with the wild-type (WT) or mutant (MUT) luciferase reporter plasmid, along with miR-185-5p mimics, inhibitors, or negative control (NC), using Lipofectamine 3000 (Life Technologies Corporation, Carlsbad, CA, USA). After incubating for 48 hours, the Promega Dual-Luciferase assay was employed to measure the firefly and Renilla enzyme activity. Creating vectors for overexpressing or silencing the HIF-1α transcription factor, as well as wild-type or mutant MKLN1-AS sequences, is essential for conducting the combination test. The procedure remains unchanged. The relative luciferase activities were determined by subtracting the Renilla luciferase activities from the firefly luciferase activities. The analysis was conducted in triplicate for each group.

### Statistical analysis

The mean ± SME of the data obtained from three triplicated independent experiments was reported. Data analysis was performed with the assistance of GraphPad Prism software (Intuitive programmer for Science, San Diego, CA) and SPSS 26.0 (SPSS Inc., Chicago, IL, USA). The comparison between two normal distribution groups was conducted using the Two-tailed Student's t-test. The disparity between the two abnormal distribution groups was analyzed by nonparametric tests. For the analysis of numerous groups, a one-way ANOVA was undertaken. Survival curves were compared using log-rank tests and depicted using the Kaplan-Meier method. Genes were analyzed for correlation using Pearson's correlation coefficient. Statistical significance was determined by using P values below 0.05 in all conducted tests.

## Result

### Identification of MKLN1-AS as a key hypoxia-responsive lncRNA in PDAC carcinogenesis

**Fig. 1:**
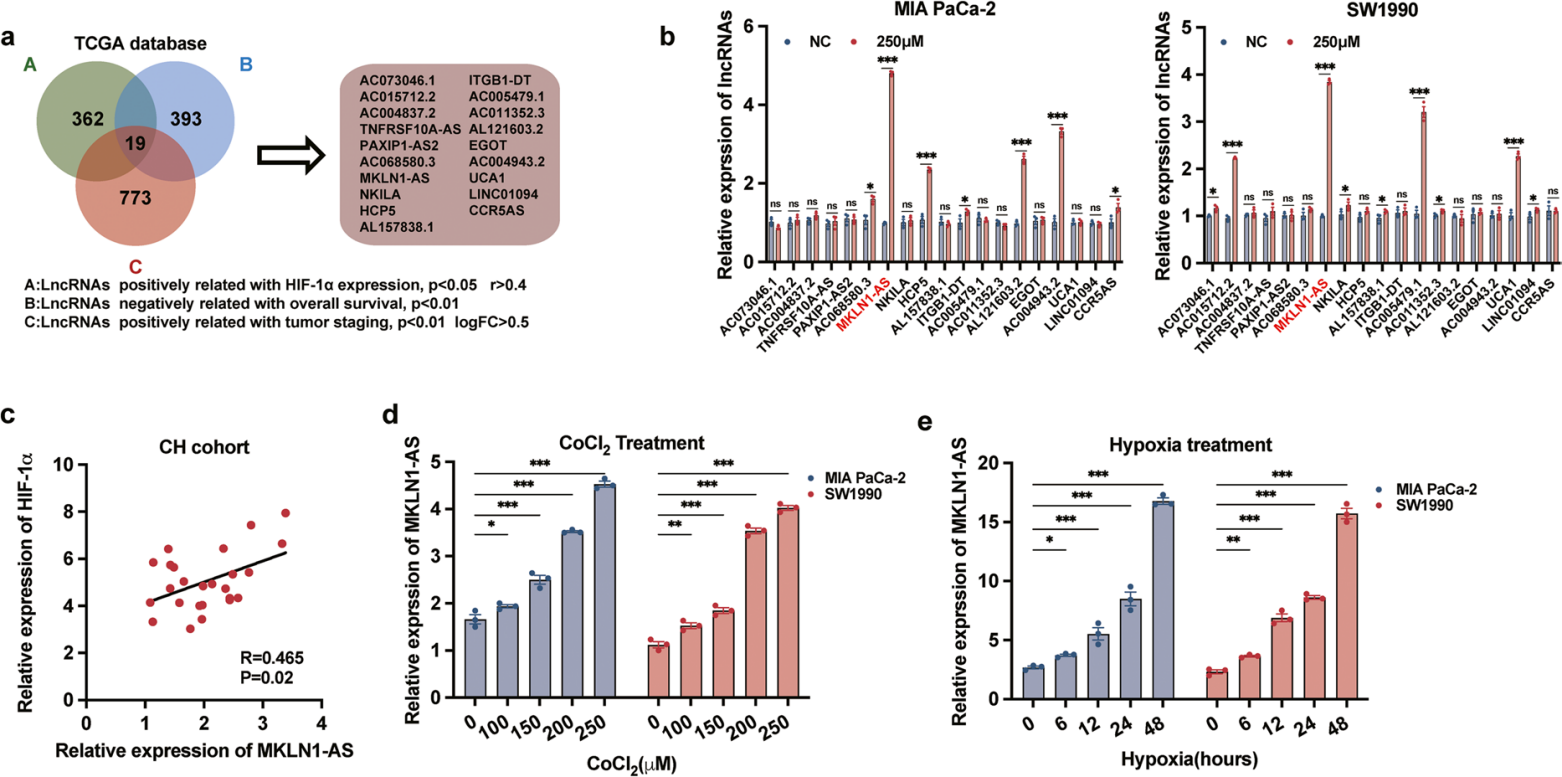
Identification of MKLN1-AS as a key hypoxia-responsive lncRNA in pancreatic carcinogenesis. **a** Three-step screening strategy: *A*. lncRNAs that exhibited a significantly positive correlation with the expression of HIF-1α (*p* < 0.05, *r* > 0.4); *B*. lncRNAs that displayed a negative association with overall survival (*p* < 0.01), *C*. lncRNAs with a positive correlation with tumor staging (logFC > 0.5, *p* < 0.01). **b** qRT-PCR analysis 19 lncRNAs expreesion in MIA PaCa-2 cells and SW1990 cells following of CoCl_2_ (250 μM) treatment. **c** qRT-PCR analysis correlation between MKLN1-AS and HIF-1α in the CH cohort (*p* = 0.02, *N*=25). **d** qRT-PCR analysis of MKLN1-AS expression in PDAC cells treated with 0, 100, 150, 200, or 250 μM CoCl_2_. **e** qRT-PCR analysis of MKLN1-AS expression in PDAC cells under hypoxia treatment (1% O_2_) for 0, 6, 12, 24, and 48 hours. Data are means ± SEM and are representative of at least 3 independent experiments. (*P≤0.05, **P≤0.01, and ***P≤0.001. NS, not significant)

To identify downstream effective lncRNAs to HIF-1α (i.e., hypoxia-responsive lncRNAs), we conducted an analysis of mRNA expression profiles from the TCGA database of PDAC. A three-step screening strategy was used: 1. lncRNAs showing a significant positive correlation with HIF-1α expression, requiring a p-value below 0.05 and a correlation coefficient (r) above 0.4; 2. lncRNAs that displayed a negative association with overall survival, with a p-value less than 0.01; 3. lncRNAs correlating positively with cancer staging, having a logFC above 0.5 and a p-value below 0.01. In total, 19 lncRNAs met these criteria and were thus designated as candidate hypoxia-responsive lncRNAs (Fig. 1a).

CoCl_2_ is a well-established activator of HIF-1α (Kong et al. 2017; Lv et al. 2018). We further treated two PDAC cell lines (MIA PaCa-2 and SW1990) with 250 μM CoCl_2_ for 24 hours. Confirmation of HIF-1α activation was achieved through qRT-PCR and western blot analysis (Supplementary Fig. 1a, b). Only one lncRNA, named MKLN1-AS, was consistently upregulated in both cell lines (Fig. 1b). Additionally, AC004943.2 and AL121603.2 were upregulated in Mia PaCa-2, while UCA1 and AC005479.1 were upregulated in SW1990. To examine the direct regulation of HIF-1α on these lncRNAs, we established HIF-1α-overexpressed and -knockdown cell lines (MIA PaCa-2 and SW1990) respectively. The transfection efficiency was confirmed through qRT-PCR and western blot analysis (Supplementary Fig. 1c, d). Expression of MKLN1-AS changed accordingly with the enforced or decreased levels of HIF-1α, with the other lncRNAs did not (Supplementary Fig. 1e). Positive correlation between HIF-1α and MKLN1-AS was identified in a tissue cohort with 25 PDAC cases from Changhai hospital (Fig. 1c, *P* = 0.02, Supplementary Table 1). In addition, we examined whether MKLN1-AS expression could be induced by hypoxia by treating PDAC cells with CoCl_2_. The levels of MKLN1-AS expression in MIA PaCa-2 and SW1990 cells were elevated with increasing treatment doses, as shown in Fig. 1d. MKLN1-AS expression significantly rose in PDAC cells when MIA PaCa-2 and SW1990 were subjected to hypoxia (1% O2) for 48 hours (Fig. 1e). Collectively, these findings identified MKLN1-AS is a key hypoxia-responsive lncRNA that is significantly involved in HIF-1α-mediated PDAC carcinogenesis.

### MKLN1-AS is highly expressed in PDAC and negatively associated with prognosis

**Fig. 2:**
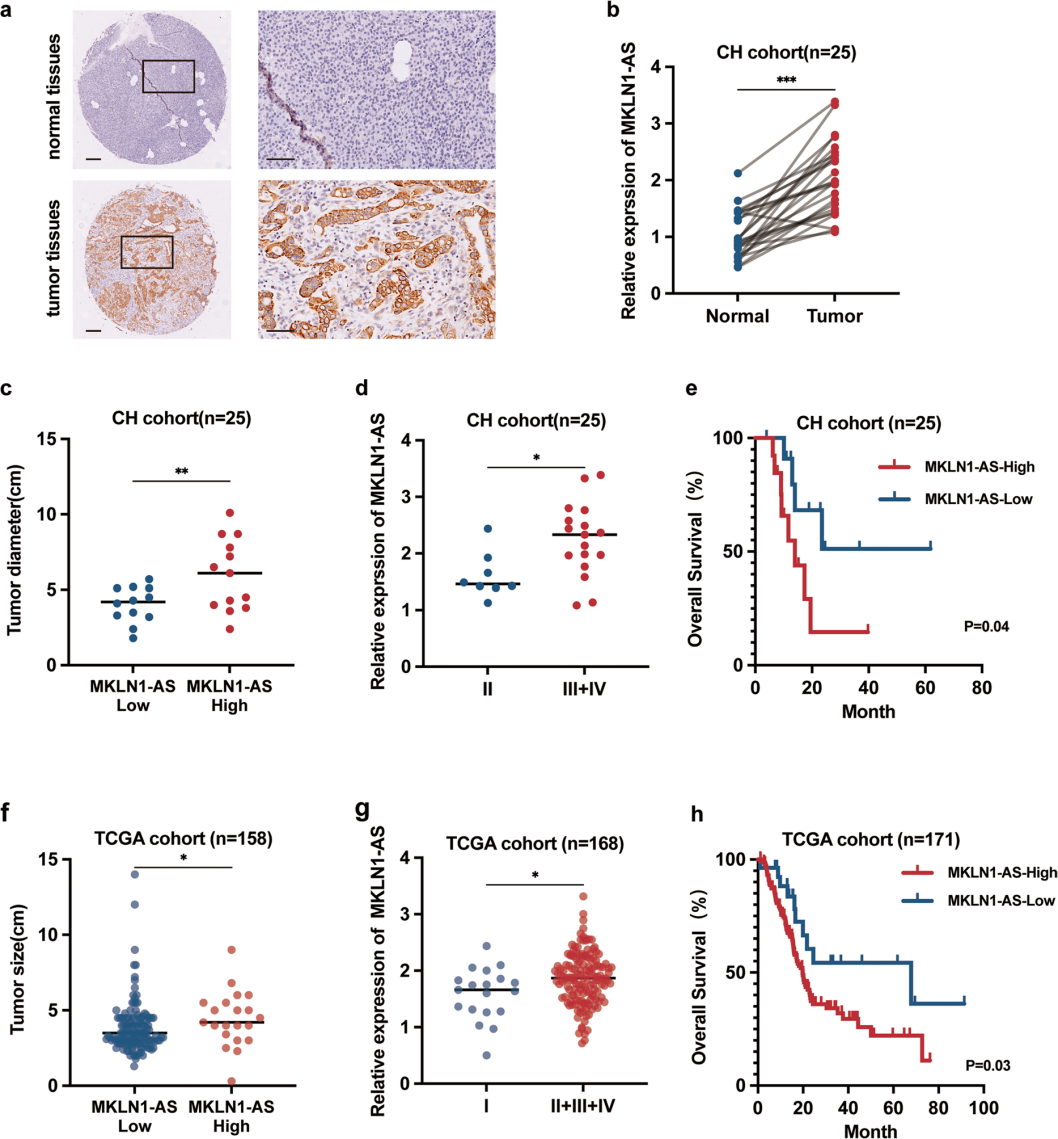
MKLN1-AS is highly expressed in PDAC and negatively associated with prognosis. **a** In situ hybridization (ISH) analysis of MKLN1-AS was performed in paired PDAC tissues and adjacent tissues (CH cohort). Representative images are shown for expression of MKLN1-AS. Scale bars, 100 μm. **b** qRT-PCR analysis of MKLN1-AS was performed in 25 paired PDAC tissues and adjacent tissues (CH cohort). MKLN1-AS expression level was normalized to that of β-actin. **c** Correlation between MKLN1-AS expression and tumor diameter in the CH cohort. **d** Correlation between MKLN1-AS expression and tumor differentiation grades in the CH cohort. **e** Kaplan-Meier survival analysis showing the impact of MKLN1-AS expression on overall survival in PDAC patients from the CH cohort (*p*=0.04). Clinical data and gene expression data from TCGA database were accessible at https://portal.gdc.cancer.gov/ with quality-controlled, derived from 171 PDAC patients. **f** Correlation between MKLN1-AS expression and tumor size in the TCGA cohort. **g** Correlation between MKLN1-AS expression and tumor differentiation grades in the TCGA cohort. **h** Illustration of the association between high MKLN1-AS expression and poor prognosis in PDAC patients within the TCGA cohort. Data are means ± SEM. (*P≤0.05, **P≤0.01, and ***P≤0.001)

We conducted qRT-PCR to determine MKLN1-AS expression levels in 25 pairs of PDAC and para-cancerous samples (CH cohort). In comparison with para-cancerous tissues, PDAC tissues exhibited a significant upregulation of MKLN1-AS performed by in situ hybridization (ISH) and qPCR assay (Fig. 2a, b, *P*<0.001). In addition, increased levels of MKLN1-AS were associated with greater tumor size (Fig. 2c, *P*<0.01), poorer differentiation grades (Fig. 2d, *P*<0.05), and shorter overall survival (OS) (Fig. 2e, *P* = 0.04). We further delved into transcriptome sequencing data obtained from 171 PDAC patients in the TCGA database (Supplementary Table 2). Consistent with our results in CH cohort, expression of MKLN1-AS expression was elevated in larger tissues ( Fig. 2f, *P*<0.01), advanced histological grade (Fig. 2g, *P*<0.01), and shorter survival (Fig. 2h, *P* = 0.03). These findings suggest that MKLN1-AS could potentially serve as a marker for PDAC and may play a vital role in pancreatic carcinogenesis.

### MKLN1-AS promoted PDAC development in vitro and in vivo

**Fig. 3:**
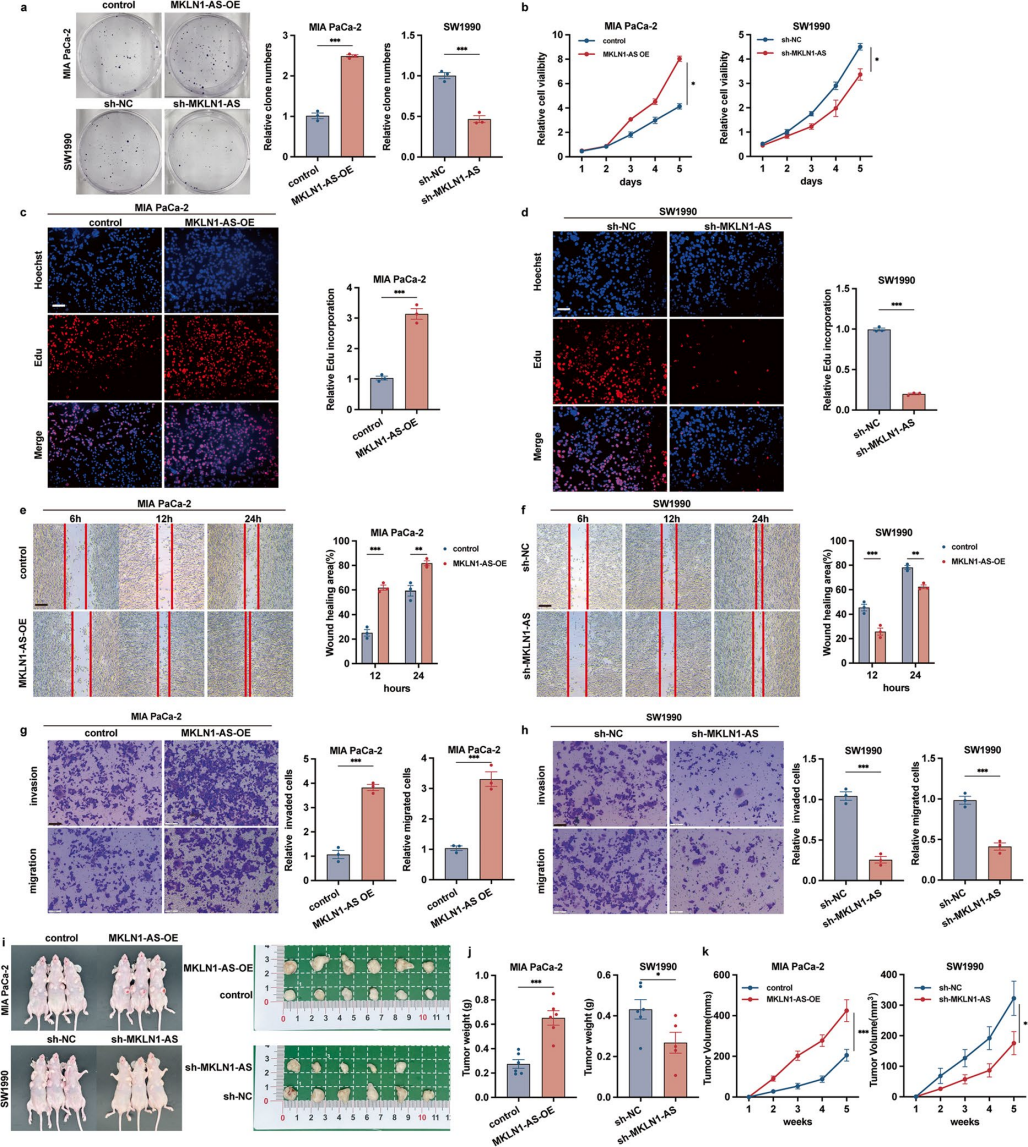
MKLN1-AS promoted PDAC development in vitro and in vivo. **a** Representative image of colony formation assay of overexpressed MKLN1-AS MIA PaCa-2 and knockdown MKLN1-AS SW1990. **b** CCK-8 assay of MKLN1-AS overexpressed MIA PaCa-2 and MKLN1-AS knockdown SW1990. At the indicated time points, the number of cells per well was measured by the absorbance (450 nm). **c, d** Representative image, and quantification analysis of Edu assay of MKLN1-AS overexpressed MIA PaCa-2 and MKLN1-AS knockdown SW1990. Scale bar: 100 μm. Representative image of scratch wound assay (Scale bar: 250 μm) **(e, f)** and Transwell **(g, h)**, quantification analysis assays of overexpressed MKLN1-AS MIA PaCa-2 and knockdown MKLN1-AS SW1990. Scale bar: 100 μm. MKLN1-AS-overexpressing MIA PaCa-2 or MKLN1-AS-knockdown SW1990 cells were subcutaneous injections in the anterior armpit of nude mice (1 × 10^6^ cells per mouse, six mice per group). **i** Gross tumors, tumor weight (**j**) and tumor volume (**k**) from indicated groups were shown. The formula for calculating tumor volume being ab^2^/2, where *a* is the longest tumor diameter and *b* is the perpendicular diameter. Data are means ± SEM and are representative of at least 3 independent experiments. (*P≤0.05, **P≤0.01, and ***P≤0.001. NS, not significant)

Levels of MKLN1-AS expression were subsequently analyzed in both normal (HPNE) and cancerous pancreatic duct epithelial cells. Elevated levels of MKLN1-AS were identified throughout all PDAC cell lines in comparison with HPNE (Supplementary Fig. 2a). To examine the biofunctions of MKLN1-AS, MKLN1-AS-overexpressed and -knockdown cell lines were constructed in MIA PaCa-2 and SW1990 cell lines respectively, and transfection efficacy was validated by qRT-PCR (Supplementary Fig. 2b, c). shMKLN1-AS#2 was selected for following experiments based on its superior knockdown efficiency. The experiments using Cell-Counting Kit-8 test, Colony Formation, and Edu assay revealed that enforced expression of MKLN1-AS promoted proliferation of MIA PaCa-2 while knocking down of MKLN1-AS did the opposite in SW1990 cells (Fig. 3a-d). Because increased MKLN1-AS expression correlated with advanced PDAC TNM staging (Fig. 2d, g), we further investigated the role of MKLN1-AS in PDAC cells migration and invasion. Wound healing and transwell assay showed that elevated MKLN1-AS increased, and attenuated MKLN1-AS decreased the migratory and invasion capabilities of PDAC cells (Fig. 3e-h), indicating that MKLN1-AS is potentially involved in PDAC metastasis.

Considering the observed MKLN1-AS enhancement cells proliferation, migration, and invasion in vitro by MKLN1-AS, we conducted an investigation to ascertain the tumorigenic potential of MKLN1-AS utilizing xenograft mouse models. For the purpose of assessing tumor growth, we introduced PDAC cells either overexpressiong or with knockdown of MKLN1-AS subcutaneously into 5-week-old mice. Our findings demonstrated the transfection of MKLN1-AS significantly promoted the subcutaneous growth of MIA PaCa-2, whereas the knockdown of MKLN1-AS elicited the converse effect on SW1990 (Fig. 3i-k). These data further substantiate the essential role of MKLN1-AS in the development of PDAC.

### MKLN1-AS mediated the promotive effects of HIF-1α on PDAC development

**Fig. 4:**
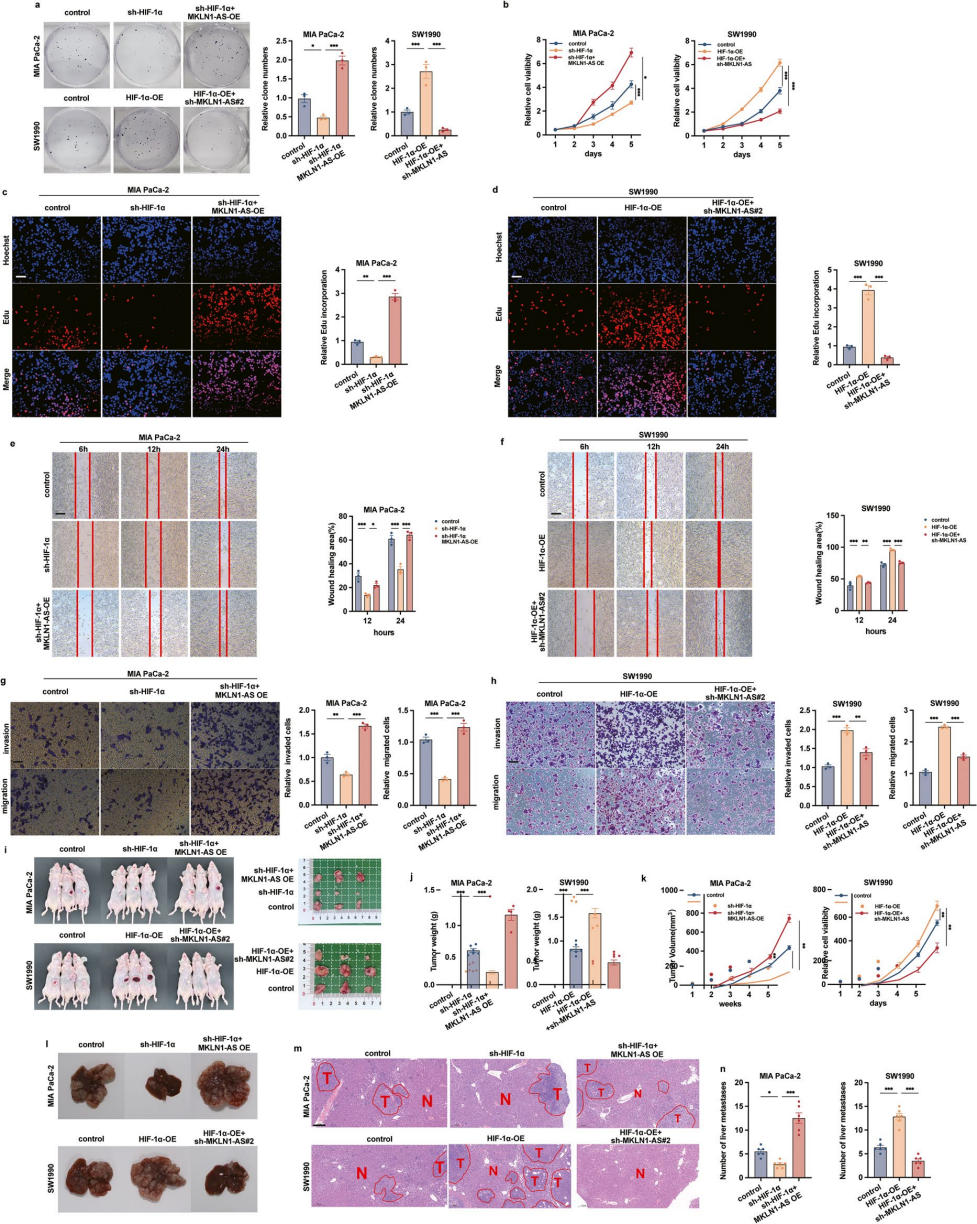
MKLN1-AS mediated the promotive effects of HIF-1α on PDAC development. **a** Representative images of colony formation assay in HIF-1α-knockdown MIA PaCa-2 cells with MKLN1-AS overexpression and HIF-1α-overexpressed SW1990 cells with MKLN1-AS knockdown. **b** In vitro assessment of PDAC cell growth using the cell counting kit-8 (CCK-8) at the specified time points. **c, d** Representative Images and quantification analysis of Edu Assay in PDAC Cells. Results were observed under a fluorescence microscope, and relative light intensity was quantified using ImageJ software. Scale bar: 100 μm. **e, f** Representative Images and quantification Analysis of scratch wound assays (Scale bar: 250 μm) and transwell assays (Scale bar: 100 μm) in PDAC Cells. **g, h** Migration or invasion quantification and mean migration or invasion area (mm^2^) were determined using ImageJ analysis software (version 1.53). HIF-1α-knockdown MIA PaCa-2 cells with MKLN1-AS overexpression and HIF-1α-overexpressed SW1990 cells with MKLN1-AS knockdown were subcutaneously injected into six nude mice per group at a concentration of 1 × 10^6^ cells per mouse. Gross tumors (**i**), tumor weights (**j**), and tumor volume (**k**) are presented. **l, m** Representative images of gross liver metastasis and HE-stained sections of metastatic nodules in the liver of nude mice (tumor areas are outlined with lines: T, tumor; N, normal). Scale bars: 200 μm. **m** Means and standard deviation of the numbers of liver metastases were calculated. Data are means ± SEM and are representative of at least 3 independent experiments. (*P≤0.05, **P≤0.01, and ***P≤0.001. NS, not significant)

As our work identified that MKLN1-AS as an important hypoxia-responsive lncRNA, we further tested if MKLN1-AS was involved in the promotive effects of HIF-1α on PDAC. As shown in Fig. 4a-d, overexpression of MKLN1-AS significantly attenuated the inhibitory effects on MIA PaCa-2 cells by HIF-1α knockdown. In parallel, deletion of MKLN1-AS abolished the effects of HIF-1α in promoting cell proliferation (Fig. 4a-d). Scratch wound (Fig. 4e, f) and transwell (Fig. 4g, h) assays corroborated the parallel role of MKLN1-AS on HIF-1α-induced pro-tumor biofunctions.

We further conducted *in vivo* experiments to explore the involvement of MKLN1-AS in mediating the promotive effects of HIF-1α on tumor growth and metastasis. Increased levels of MKLN1-AS significantly reduced the inhibitory effects of sh-HIF-1α on MIA PaCa-2 cells, whereas the absence of MKLN1-AS eliminated the stimulatory effects of HIF-1α on SW1990 tumor growth (Fig. 4i-k).

To assess the metastatic potential of these tumors, we intravenously inject tumor cells into mice through the ileocolic vein. Enhanced expression of MKLN1-AS significantly counteracted the suppressive effects of sh-HIF-1α on hepatic metastasis in MIA PaCa-2 cells (Fig. 4l-n). Consistently, deletion of MKLN1-AS nullified the effects of HIF-1α on promoting hepatic metastasis in SW1990 cells. These data collectively identify the involvement of MKLN1-AS in mediating the promotive effects of HIF-1α on PDAC development.

### MKLN1-AS promoted PDAC progression via elevated TEAD1 expression

**Fig. 5:**
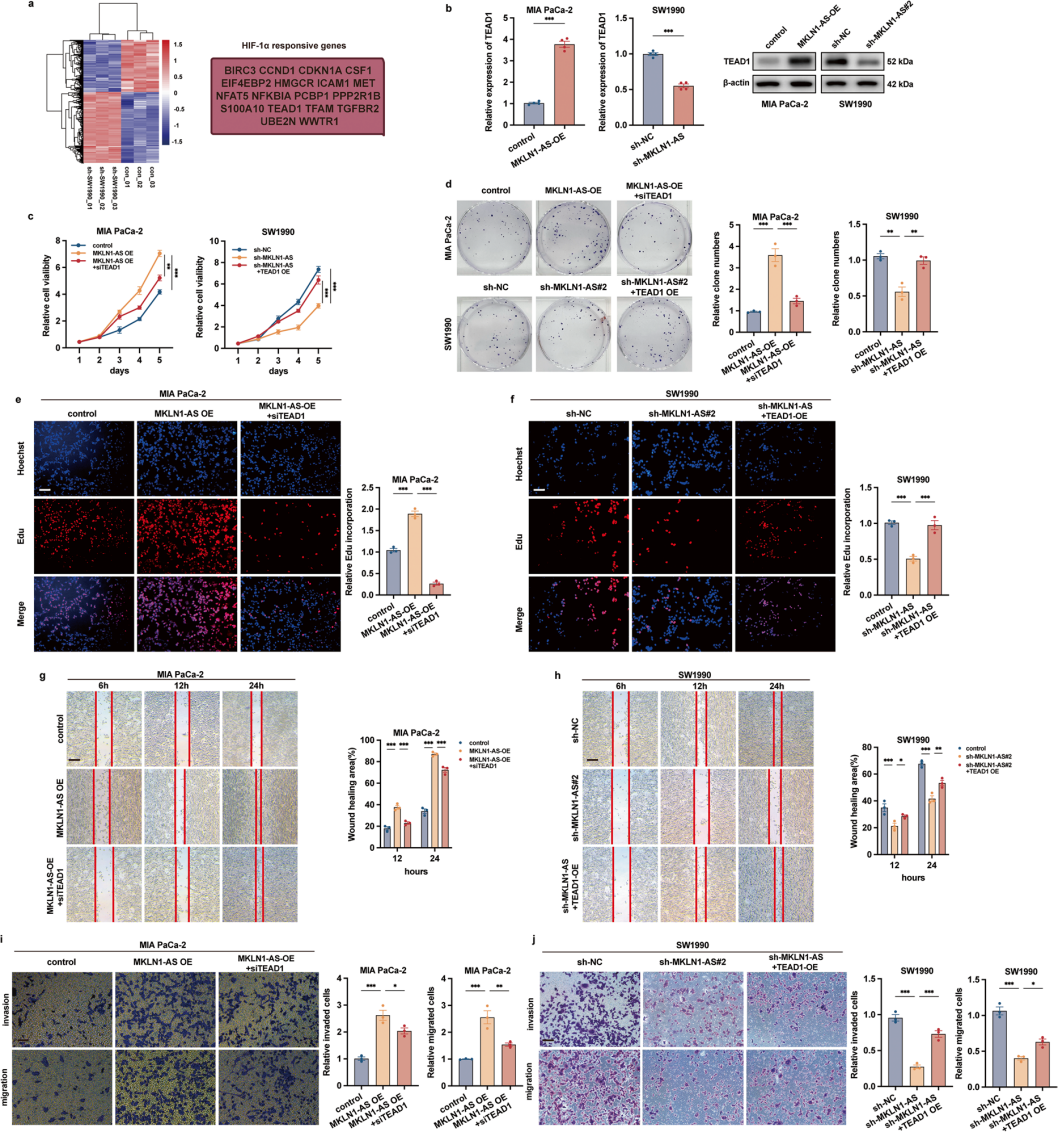
MKLN1-AS promoted PDAC progression via elevated TEAD1 expression. **a** Illustration of target genes regulated by MKLN1-AS and correlated with HIF-1α. **b** Expression of TEAD1 at mRNA and protein levels in MKLN1-AS-overexpressed MIA PaCa-2 and MKLN1-AS-knockdown SW1990 Cells. **c** CCK-8 Assay demonstrating the role of TEAD1 in mediating MKLN1-AS-induced proliferation of PDAC cells. Representative images and quantification analysis of colony formation (**d**) and edu assay (**e, f**) in HIF-1α-knockdown MIA PaCa-2 cells with MKLN1-AS overexpression and HIF-1α-overexpressed SW1990 cells with MKLN1-AS knockdown. Scale bar: 100 μm in the Edu assay. Wound healing (**g, h**) and transwell assay (**i, j**) results for MIA PaCa-2 cells induced by MKLN1-AS with or without TEAD1 shRNA transfection and SW1990 cells silenced by MKLN1-AS with or without TEAD1 transfection. Scale bar: 250μm for wound healing assay, 100 μm for transwell assay. Data are means ± SEM and are representative of at least 3 independent experiments. (*P≤0.05, **P≤0.01, and ***P≤0.001. NS, not significant).

To investigate the target genes and relevant signaling pathways regulated by MKLN1-AS, we compared the gene expression profiles of SW1990 cells transfected with shMKLN1-AS to those of the control group. Deletion of MKLN1-AS was validated by qRT-PCR. Our analysis revealed that 44 genes were significantly downregulated in SW1990 cells with shMKLN1-AS (log2 fold change <-1, q < 0.01). Of the 44 genes mentioned above, 36 genes displayed significant correlations with HIF-1α by collecting data from Genecards public database, and we selected the top 50% of these genes based on their relevance scores for further analysis (Fig. 5a). In subsequent investigations, we conducted co-expression studies for these 18 genes with HIF-1α using two datasets: TCGA, which included 171 PDAC patients, and GEO dataset (GSE57495), consisting of 63 PDAC patients. Remarkably, we found that TEAD1, TGFBR2, and WWTR1 consistently exhibited statistically significant correlations with HIF-1α expression in both datasets (as shown in Supplementary Fig. 3a and 3b).

TEAD1, a crucial element of the Hippo signaling pathway, is vital for regulating the biological activities of YAP (Sun et al. 2021; Zhao et al. 2008). We further explored if TEAD1 was potentially involved in MKLN1-AS mediated development of PDAC. The data we collected indicated a positive correlation between MKLN1-AS and TEAD1 in PDAC tissues from the CH cohort, with a correlation coefficient of 0.468 and a significance level below 0.05 (Supplementary Fig. 3c). The data from TCGA cohort consistently identified the positive correlation between TEAD1 and MKLN1-AS (*R* = 0.286, *P*<0.001) (Supplementary Fig. 3d). Analysis of survival using TCGA data revealed a significant link between elevated TEAD1 expression and decreased overall survival (*P*=0.02) (Supplementary Fig. 3e). MKLN1-AS was found to promote TEAD1 expression in PDAC cells by western blotting and qRT-PCR, whereas knocking down MKLN1-AS inhibited TEAD1 expression (Fig. 5b).

Next, we investigated the role of TEAD1 in the impacts of MKLN1-AS on tumor cell aggressiveness. As illustrated in Fig. 5c-f, knocking down TEAD1 in MIA PaCa-2 cells abrogated the effects of MKLN1-AS on promoting cell proliferation. On the contrary, overexpressing TEAD1 notably reversed the growth inhibition in SW1990 cells induced by the knockdown of MKLN1-AS. We further tested if TEAD1 was involved in the promotive effects of MKLN1-AS on PDAC migration and invasion. Our results showed that deletion of TEAD1 abrogate the effects of MKLN1-AS on promoting cell migration and invasion (Fig. 5g, i), whereas overexpression of TEAD1 significantly attenuated the inhibitory effects on MIA PaCa-2 cells by MKLN1-AS knockdown (Fig. 5h, j). These findings collectively identified that the promotive effects on PDAC by MKLN1-AS were at least partially dependent on TEAD1.

### MKLN1-AS functions as a miRNA sponge for miR-185-5p to regulate the expression of TEAD1

**Fig. 6:**
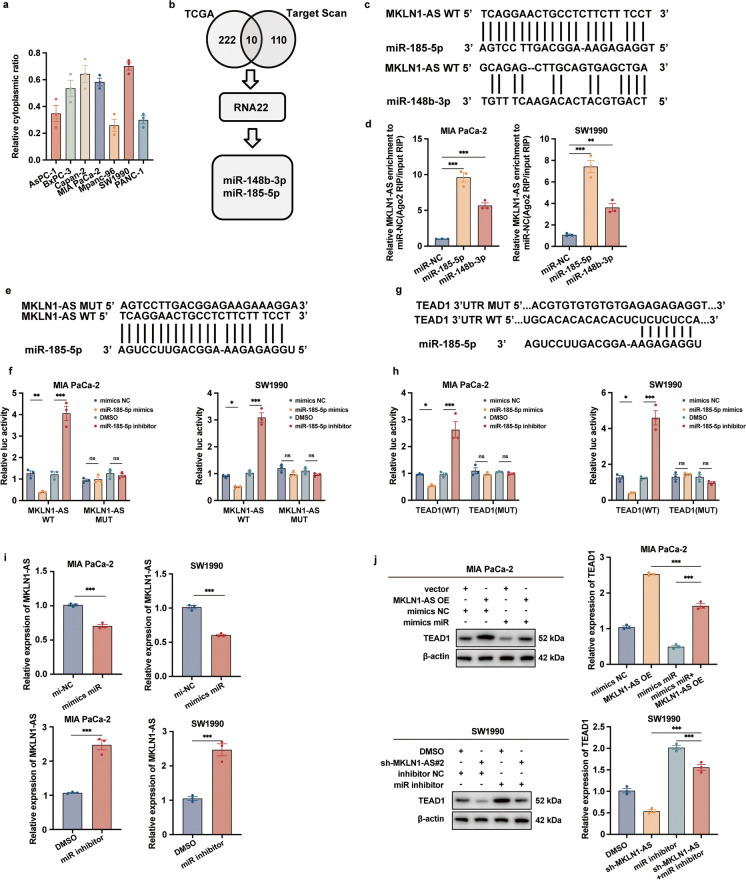
MKLN1-AS function as a miRNA sponge for miR-185-5p to regulate the expression of TEAD1. **a** Subcellular distribution analysis of MKLN1-AS by qRT-PCR. **b** Flowchart illustrating our screening strategy. The expression of 222 miRNAs positively associated with the overall survival of PDAC patients from the TCGA cohort. 110 miRNAs were identified in both MKLN1-AS and TEAD1 3’-UTR using Target Scan. RNA22 v2.0 identified two miRNAs (miR-148b-3p and miR-185-5p) targeting MKLN1-AS and TEAD1 3’UTR. **c** Predicted binding sites of miR-148b-3p and miR-185-5p in the MKLN1-AS transcript. **d** Ago2-RIP assays demonstrating the enrichment of MKLN1-AS in cells transfected with miR-NC or predicted miRNAs. **e** Sequence of MKLN1-AS WT and MUT. **f** Dual-luciferase assays implied that MKLN1-AS-WT instead of MKLN1-AS-Mut was targeted by miR-185-5p. **g** Predicted binding sites of miR-185-5p in the TEAD1 3’UTR, showing mutant nucleotide sequences of the target site. **h** Dual-luciferase assays implied that TEAD1 3’UTR-WT instead of TEAD1 3’UTR-Mut was targeted by miR-185-5p. **i** Analysis of MKLN1-AS expression transfected with specific miR-185-5p inhibitors or mimics. **j** Analysis of TEAD1 mRNA and protein levels in MKLN1-AS-overexpressed MIA PaCa-2 cells transfected with specific miR-185-5p mimics and MKLN1-AS-knockdown SW1990 cells transfected with miR-185-5p inhibitors. Data are means ± SEM and are representative of at least 3 independent experiments. (*P≤0.05, **P≤0.01, and ***P≤0.001. NS, not significant).

To determine the subcellular localization of MKLN1-AS, we utilized a cellular fractionation assay and then conducted qRT-PCR analysis. The results, as depicted in Fig. 6a, indicated that MKLN1-AS primarily resides in the cytosol of PDAC cells, with a minor fraction present in the nucleus. Recognizing that cytoplasmic lncRNAs often function as ceRNA (Fan et al. 2018; Xu et al. 2019), we constructed a ceRNA network to establish a connection between MKLN1-AS and TEAD1 via miRNAs. Initially, we discovered miRNAs linked to the survival rate of individuals with PDAC by analyzing the TCGA group. Subsequently, we employed TargetScan and RNA22 v.2.0 to identify the shared miRNAs that can potentially bind to both MKLN1-AS and TEAD1 3’-UTRs. As illustrated in Fig. 6b, two miRNAs, miR-148b-3p and miR-185-5p, remained in the ceRNA network. The binding sites between MKLN1-AS and both miRNAs are presented in Fig. 6c.

We further conducted Ago2-RIP assay, showing miR-185-5p overexpression was significantly enriched for MKLN1-AS pulldown by Ago2. In comparison to IgG RIP, the increase in AGO2 RIP samples was eightfold (Fig. 6d). Subsequently, we designed reporter constructs with wildtype (MKLN1-AS-WT) and mutant (MKLN1-AS-Mut) miR-185-5p binding regions wihtin the MKLN1-AS sequence (Fig. 6e). miR-185-5p mimics transfection led to a notable decrease in luciferase activity in MKLN1-AS-WT reporters, while no effect was observed in MKLN1-AS-Mut reporters (Fig. 6f). In parallel, luciferase reporters were constructed with both wild-type and altered binding sites in the 3’-UTR areas of TEAD1 (Fig. 6g). Transfection with miR-185-5p mimics significantly reduced luciferase activity in reporters containing TEAD1 3’UTR-WT, but not in those with mutations (Fig. 6h). Furthermore, the decreased expression of MKLN1-AS is caused by the increased expression of miR-185-5p, whereas inhibition of miR-185-5p results in the upregulation of MKLN1-AS (Fig. 6i).

Furthermore, we explored the inhibition of TEAD1 by miR-185-5p in PDAC cells. As anticipated, the increase of miR-185-5p led to a reduction in TEAD1 abundance at both the protein and mRNA levels, while specific miR-185-5p inhibitors elevated TEAD1 levels in terms of protein and mRNA (Fig. 6j).

### Transcriptional regulation of MKLN1-AS expression in pancreatic cancer by HIF-1α

**Fig. 7:**
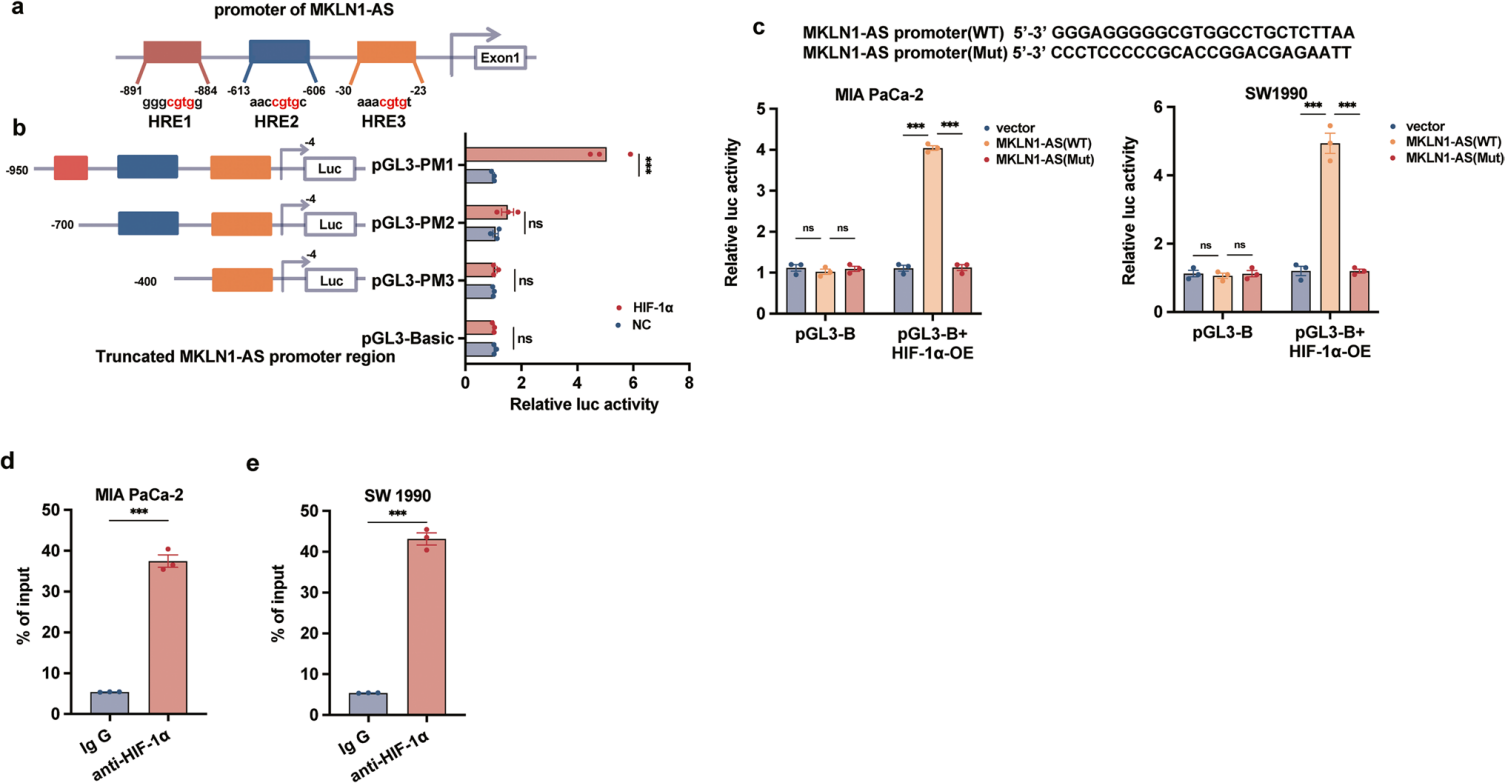
Transcriptionally regulation of MKLN1-AS expression in pancreatic cancer by HIF-1α. **a** Schematic diagram illustrating the MKLN1-AS promoter luciferase reporter constructs with HIF-1α binding to HREs. HRE: Hypoxia Response Elements. **b** Luciferase promoter assay in 293T cells demonstrating transcriptional activation of MKLN1-AS by HIF-1α through binding to HRE1. **c** Dual-luciferase assays indicated that the MKLN1-AS-WT promoter instead of MKLN1-AS-Mut, binds with HIF-1α. **d, e** ChIP assay demonstrating the enrichment of PCR fragments covering HRE1 pulled down with the HIF-1α antibody in both MIA PaCa-2 and SW1990 cells. Data are means ± SEM and are representative of at least 3 independent experiments. (*P≤0.05, **P≤0.01, and ***P≤0.001. NS, not significant)

**Fig. 8:**
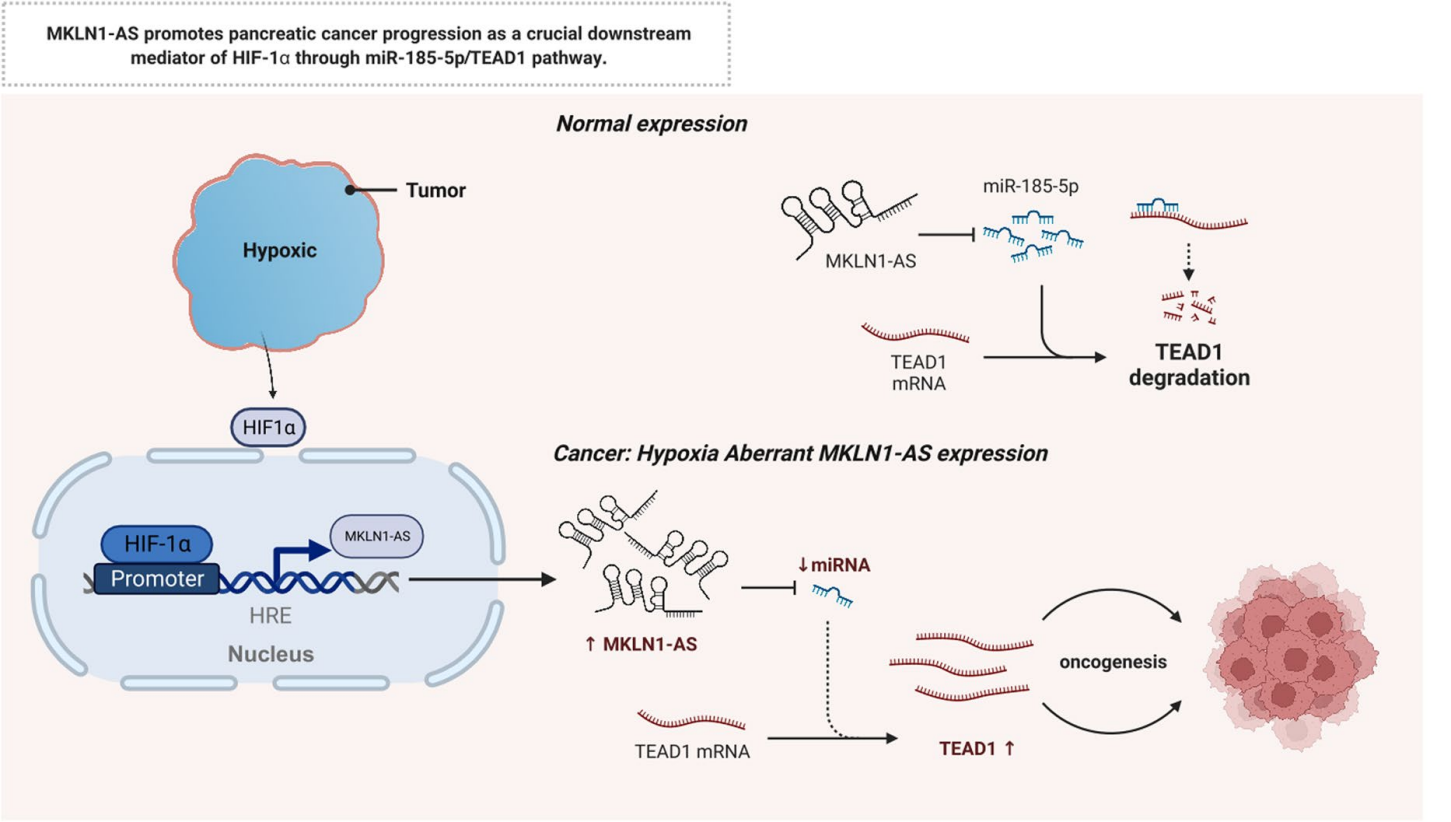
The function model underlying the mechanism of MKLN1-AS in PDAC tumorigenesis. MKLN1-AS promotes pancreatic cancer progression as a crucial downstream mediator of HIF-1a through miR-185-5p/TEAD1 pathway

Expression of MKLN1-AS changed accordingly with the enforced or decreased levels of HIF-1α (Supplementary Fig. 1d ). Additionally, overexpression or knockdown of MKLN1-AS have little effects on HIF-1α expression, indicating that regulation of MKLN1-AS by HIF-1α is unidirectional (Supplementary Fig. 4a, b). Bioinformatics analysis focused on potential hypoxia response elements (HREs) (5'-CGTG-3') in the promoter region (Kong et al. 2017; Zhao et al. 2014) of MKLN1-AS, indicating the existence of several potential HIF-1α binding locations (Supplementary Fig. 4c). Three primary HIF-1α-binding locations were discovered in the MKLN1-AS promoter area, located at -891 (HRE1), -613 (HRE2), and -30 (HRE3) base pairs from the beginning of MKLN1-AS transcription (Fig. 7a). To further validate these findings, two deletion mutants (MKLN1-AS-700, covering HRE2 and HRE3; MKLN1-AS-400, covering only HRE3) and a reporter encompassing the full-length MKLN1-AS promoter (MKLN1-AS-1009, encompassing all HREs) were constructed. These reporters, along with HIF-1α expression vectors, were introduced into 293T cells. Removing the area containing HRE1 significantly reduced the promoter function of MKLN1-AS, which was stimulated by HIF-1α, as shown in the luciferase reporter assay findings (Fig. 7b). As illustrated in Fig. 7c, heightened HIF-1α expression in MIA PaCa-2 and SW1990 cells significantly augmented MKLN1-AS promoter activity. Moreover, a ChIP assay verified that HIF-1α directly interacted with HRE1 in both MIA PaCa-2 and SW1990 cells (Fig. 7d, e). Collectively, these data indicate that the activation of MKLN1-AS is, at least in part, a consequence of HIF-1α regulation. A schematic of the underlying mechanism of MKLN1-AS in PDAC progression is presented in Fig. 8.

## Discussion

Numerous studies have reported that hypoxia-induced abnormal expression of non-coding RNAs promotes neoplasm progress. However, the specific molecular mechanism of hypoxia-related genes responsible for tumor development remained limited. HIF-1α, the primary transcription factor for responding to low oxygen levels, plays a vital role in the development of tumors, participating in processes such as blood vessel formation, red blood cell production, glucose metabolism, and inflammation (Semenza 2001). Recent studies have reported that HIF-1α responsible for lncRNA PVT1-mediated pancreatic cancer enhancing (Sun et al. 2020). A different research study found that has reported that the activation of lncRNA SLC2A1 declined the expression of HIF-1α to inhibit osteoarthritis (Guan et al. 2023). During the research, it was found that lncRNA MKLN1-AS was a molecule downstream of HIF-1α, being activated at the transcriptional level by HIF-1α. HIF-1α binds to HREs located in the MKLN1-AS promoter to promote PDAC progression. Several studies have reported that MKLN1-AS correlates with targeted drug sensitivity, and is implicated in hypoxia-responsive lncRNAs, that predict HCC outcomes. However, the role and underlying mechanism of MKLN1-AS in PDAC have not been thoroughly explored (Tang et al. 2021). In our present study, we discovered MKLN1-AS as a hypoxia-responsive lncRNA, via MKLN1-AS/miR-185-5p/TEAD1 pathway promotes PDAC progression. This finding indicated that MKLN1-AS may serve as an alternative molecule cooperating with targeting HIF-1α therapy for PDAC.

Located on chromosome 7, MKLN1-AS is a lncRNA that, along with five other lncRNAs (AC139491.2, AC145207.5, AC099850.3, AL590705.3, and AL049840.5), was initially identified as a biomarker for predicting the prognosis and response to immunotherapy in patients with Hepatocellular carcinoma HCC (Cheng et al. 2022). Furthermore, MKLN1-AS played a promoted oncogenic role in HCC induced by ferroptosis and predicted the outcomes of patients on immunotherapy (Fang et al. 2022). This research unveiled a notable increase in MKLN1-AS expression in PDAC tissues when compared to non-cancerous samples, marking the first time this has been observed. PDAC patients with higher levels of MKLN1-AS have a poorer prognosis and low differentiation grades according to clinical data. And we discovered that enforced MKLN1-AS promoted PDAC cells proliferation and metastasis in vitro and in vivo. Further research exhibited that the function of MKLN1-AS promoted PDAC development via TEAD1 upregulated. Our research is the initial documentation pinpointing MKLN1-AS as a long non-coding RNA that responds to low oxygen levels in the development of PDAC, while also explaining the process by which MKLN1-AS contributes to the progression of PDAC.

An integral component of the Hippo signaling pathway, TEAD1 is known for its involvement in processes such as tumor cell proliferation, migration, epithelial-mesenchymal transition (Li et al. 2022), and drug resistance (Wei et al. 2023). The first report TEAD1 in PDAC promotion was acting as the transcription enhancer factor bound by MSLN element to enhance MSLN expression (Hucl et al. 2007). Following that, TEAD1 causes an elevation in 5-hydroxymethylcytosine levels, leading to alterations that allow for the identification of PDAC in its early stages (Guler et al. 2020). Also, in this context, mounting research explored suppressors that inhibit the expression of TEAD1. Including short isoform (PRLR-SF) (Nie et al. 2021), Cyclin-Dependent Kinase 1 (CDK1) (Zeng et al. 2017), and Fascin protein (Lin et al. 2021) contribute to suppress cell growth and spread by reducing TEAD1 expression. However, the mechanism of upregulated TEAD1 in pancreatic cancer is referred to less frequently. Our research demonstrated the increased expression of TEAD1 regulated by MKLN1-AS and validated the role of MKLN1-AS as a ceRNA that interacts with miR-185-5p to upregulate TEAD1. In our molecular experiment, after inhibiting MKLN1-AS, the expression of TEAD1 decreased. This finding indicated that inhibiting MKLN1-AS disrupted the TEAD1 signaling pathway, offering a novel means for targeting inhibition of TEAD1 in PDAC.

Since MKLN1-AS primarily localized in the cytoplasm, we subsequently explored its potential role as a ceRNA, sequestering specific miRNAs targeting TEAD1. Our bioinformatic analysis and experiment evidence revealed miR-185-5p may be absorbed by MKLN1-AS, influencing the expression of TEAD significantly. Previous research has shown different results on the influence of miR-185-5p on cancer advancement, with contradictory functions observed as both inhibiting and promoting tumor growth (Scognamiglio et al. 2022; Değerli et al. 2020; Yu et al. 2022). A recent research discovered that miR-185-5p delivered through extracellular vesicles in the blood of individuals can serve as a marker for advanced adenoma and colorectal cancer (Shi et al. 2023). In the field of PDAC research, it has been highlighted that miR-185-5p exerts regulatory control over non-protein coding RNA, thereby fostering the progression of pancreatic cancer (Li et al. 2021). The study unveils a novel suppressive function of miR-185-5p targeting the ondogene TEAD1, suggesting its potential utility as a biomarker for predicting PDAC prognosis.

To sum up, our investigation has elucidated the upregulation of a hypoxia-responsive lncRNA known as MKLN1-AS within pancreatic cancer tissue. This lncRNA functions as an oncogenic factor, contributing to the promotion of both tumor growth and metastasis. The elevated MKLN1-AS competitively interacts with miR-185-5p, resulting in TEAD1 expression increase. This molecular cascade contributes to the aggressiveness of PDAC. Targeting the HIF-1α/MKLN1-AS/miR-185-5p/TEAD1 signaling pathway holds promise as a future treatment strategy for PDAC.

## Supplementary information

ESM 1 Supplementary Figure 1. Identification of MKLN1-AS as a key hypoxia-responsive lncRNA in pancreatic carcinogenesis. a, b Western-blot and qRT-PCR assay of the expression of HIF-1α under the treatment of 0, 200, and 250μM CoCl_2_. c, d HIF-1α expression efficiency was examined by qRT-PCR assay in MIA PaCa-2 cells and SW1990 cells transfected with HIF-1α overexpressed plasmid and siRNA. e Expression of lncRNAs in PDAC cells transfected with HIF-1α overexpressed plasmid or specific siRNA. Data are means ± SEM and are representative of at least 3 independent experiments. (*P≤0.05, **P≤0.01, and ***P≤0.001. NS, not significant). (TIF 36870 kb)

ESM 2 Supplementary Figure 2. MKLN1-AS promoted PDAC development in vitro and in vivo. a MKLN1-AS expression level was detected in normal pancreatic cells (HPNE) and pancreatic cancer cells (AsPC-1, BxPC-3, Capan-2, MIA PaCa-2, Mpanc-96, SW1990). b qRT-PCR analysis of MKLN1-AS expression in MIA PaCa-2 cells transfected with MKLN1-AS overexpression plasmid. c qRT-PCR analysis of MKLN1-AS expression in SW1990 Cells transfected with MKLN1-AS-specific shRNAs (#1, #2, and #3). Data are means ± SEM and are representative of at least 3 independent experiments. (*P≤0.05, **P≤0.01, and ***P≤0.001. NS, not significant). (TIF 9646 kb)

ESM 3 Supplementary Figure 3. MKLN1-AS promoted PDAC progression via elevated TEAD1 expression. a, b Using quality-controlled data involving 171 PDAC patients from the TCGA and 63 PDAC patients from GEO cohorts, a heatmap of the correlation between HIF-1α and potential MKLN1-AS-target genes is shown. Significant values are indicated by asterisks (*); ** *p* <0.01, * *p* <0.05. The Spearman correlation between the expression of two genes was examined. MKLN1-AS and TEAD1 expression in TEAD1 tissues from the CH (c) and TCGA (d) cohorts showed a positive correlation. (e) Kaplan-Meier survival analysis showing the impact of TEAD1 expression on overall survival in PDAC patients from the TCGA cohort (*p*=0.02). (TIF 44141 kb)

ESM 4 Supplementary Figure 4. Transcriptionally regulation of MKLN1-AS expression in pancreatic cancer by HIF-1α. a, b Western-blot and RT-qPCR assay of the expression of HIF-1α with MKLN-AS overexpressed vector or MKLN1-AS knockdown siRNA. c CHIP-seq data obtained from the Cistrom database, HIF-1α ChIP-seq technique was employed to investigate the binding interactions between the promoter region of the MKLN1-AS and the HIF-1α in cancer cells. Data are means ± SEM and are representative of at least 3 independent experiments. (*P≤0.05, **P≤0.01, and ***P≤0.001. NS, not significant). (TIF 17645 kb)

ESM 5 (DOCX 23 kb)

ESM 6 (PDF 3205 kb)

## Data Availability

The datasets used or analyzed during the current study are available from the corresponding author upon reasonable request.
