# Supplementary figures and images for: MKLN1-AS promotes pancreatic cancer progression as a crucial downstream mediator of HIF-1α through miR-185-5p/TEAD1 pathway
Source: Cell Biol Toxicol. 2024 May 13;40(1):30. doi: 10.1007/s10565-024-09863-8 (PMC11090931; doi:10.1007/s10565-024-09863-8)

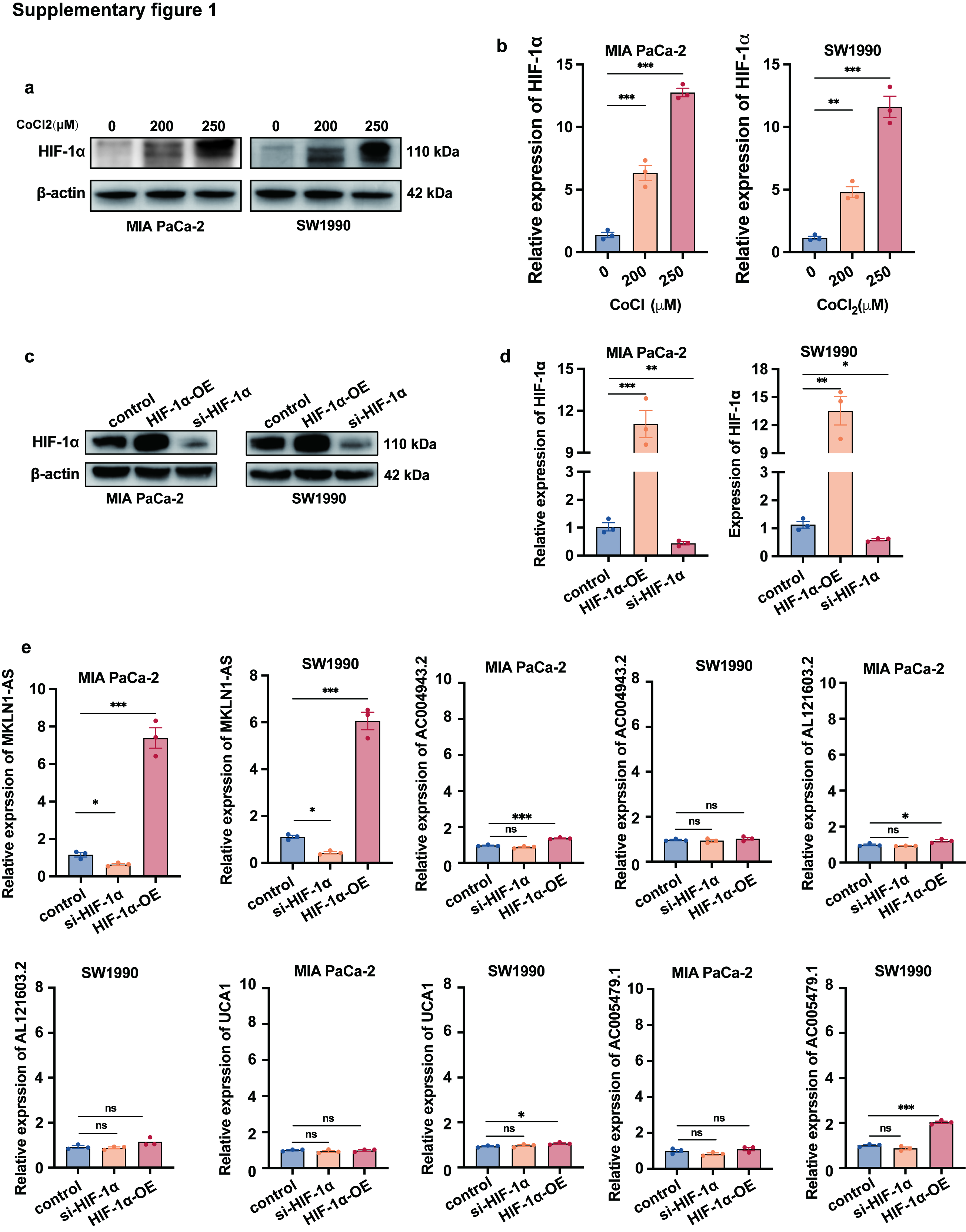

Supplement: Supplementary file 1 — Supplementary Figure 1. Identification of MKLN1-AS as a key hypoxia-responsive lncRNA in pancreatic carcinogenesis. a, b Western-blot and qRT-PCR assay of the expression of HIF-1α under the treatment of 0, 200, and 250μM CoCl2. c, d HIF-1α expression efficiency was examined by qRT-PCR assay in MIA PaCa-2 cells and SW1990 cells transfected with HIF-1α overexpressed plasmid and siRNA. e Expression of lncRNAs in PDAC cells transfected with HIF-1α overexpressed plasmid or specific siRNA. Data are means ± SEM and are representative of at least 3 independent experiments. (*P≤0.05, **P≤0.01, and ***P≤0.001. NS, not significant). (TIF 36870 kb) [file 10565_2024_9863_MOESM1_ESM.tif]

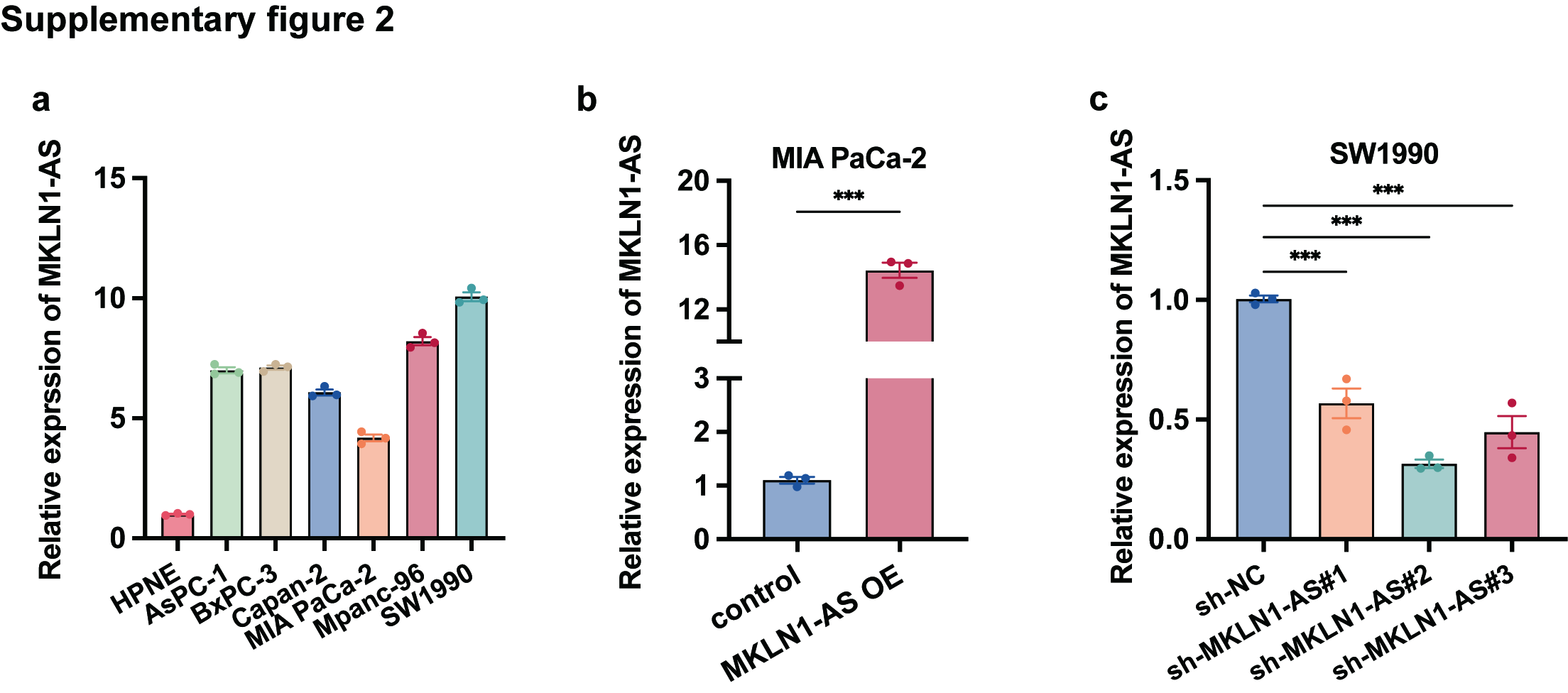

Supplement: Supplementary file 2 — Supplementary Figure 2. MKLN1-AS promoted PDAC development in vitro and in vivo. a MKLN1-AS expression level was detected in normal pancreatic cells (HPNE) and pancreatic cancer cells (AsPC-1, BxPC-3, Capan-2, MIA PaCa-2, Mpanc-96, SW1990). b qRT-PCR analysis of MKLN1-AS expression in MIA PaCa-2 cells transfected with MKLN1-AS overexpression plasmid. c qRT-PCR analysis of MKLN1-AS expression in SW1990 Cells transfected with MKLN1-AS-specific shRNAs (#1, #2, and #3). Data are means ± SEM and are representative of at least 3 independent experiments. (*P≤0.05, **P≤0.01, and ***P≤0.001. NS, not significant). (TIF 9646 kb) [file 10565_2024_9863_MOESM2_ESM.tif]

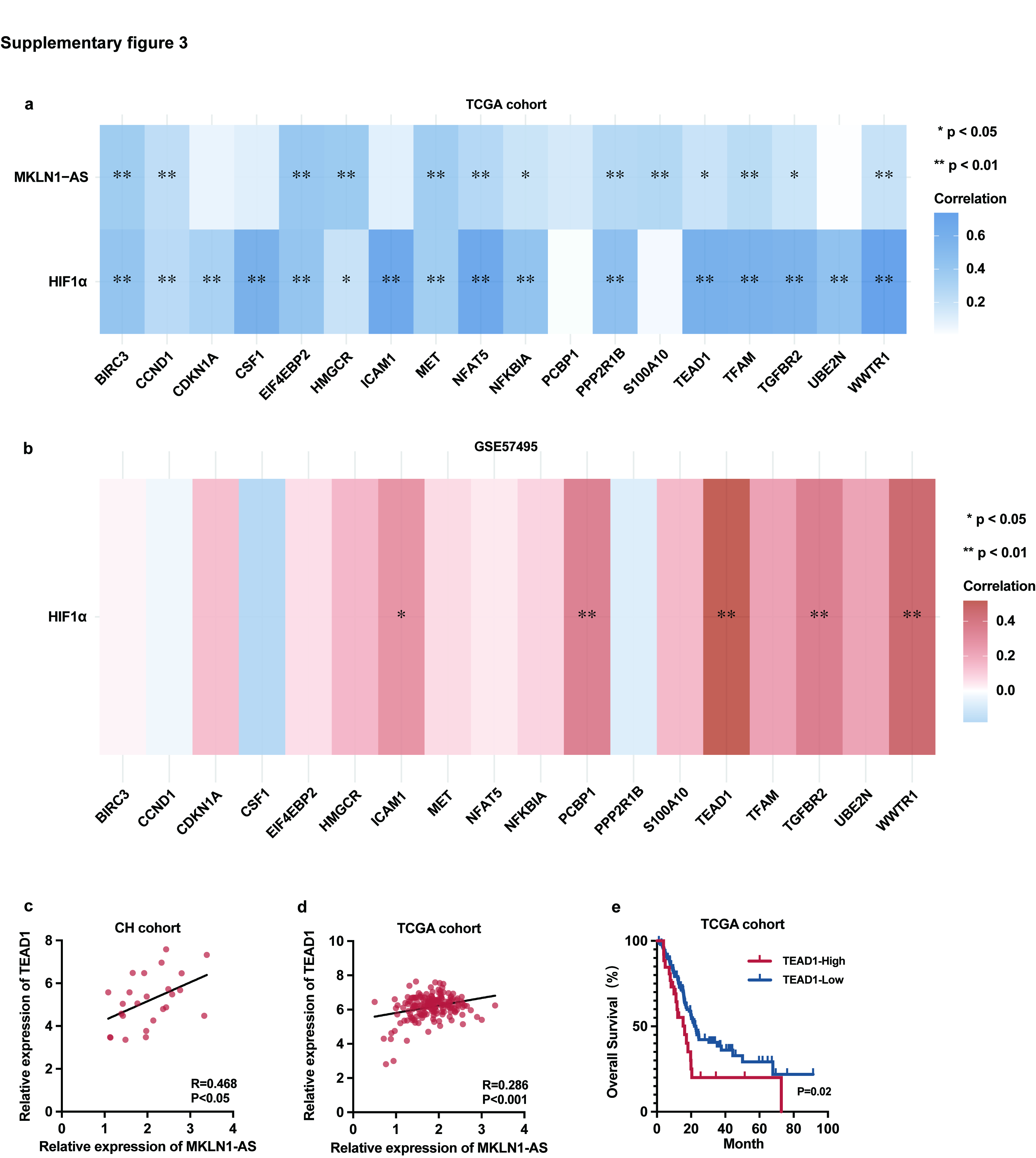

Supplement: Supplementary file 3 — Supplementary Figure 3. MKLN1-AS promoted PDAC progression via elevated TEAD1 expression. a, b Using quality-controlled data involving 171 PDAC patients from the TCGA and 63 PDAC patients from GEO cohorts, a heatmap of the correlation between HIF-1α and potential MKLN1-AS-target genes is shown. Significant values are indicated by asterisks (*); ** p <0.01, * p <0.05. The Spearman correlation between the expression of two genes was examined. MKLN1-AS and TEAD1 expression in TEAD1 tissues from the CH (c) and TCGA (d) cohorts showed a positive correlation. (e) Kaplan-Meier survival analysis showing the impact of TEAD1 expression on overall survival in PDAC patients from the TCGA cohort (p=0.02). (TIF 44141 kb) [file 10565_2024_9863_MOESM3_ESM.tif]

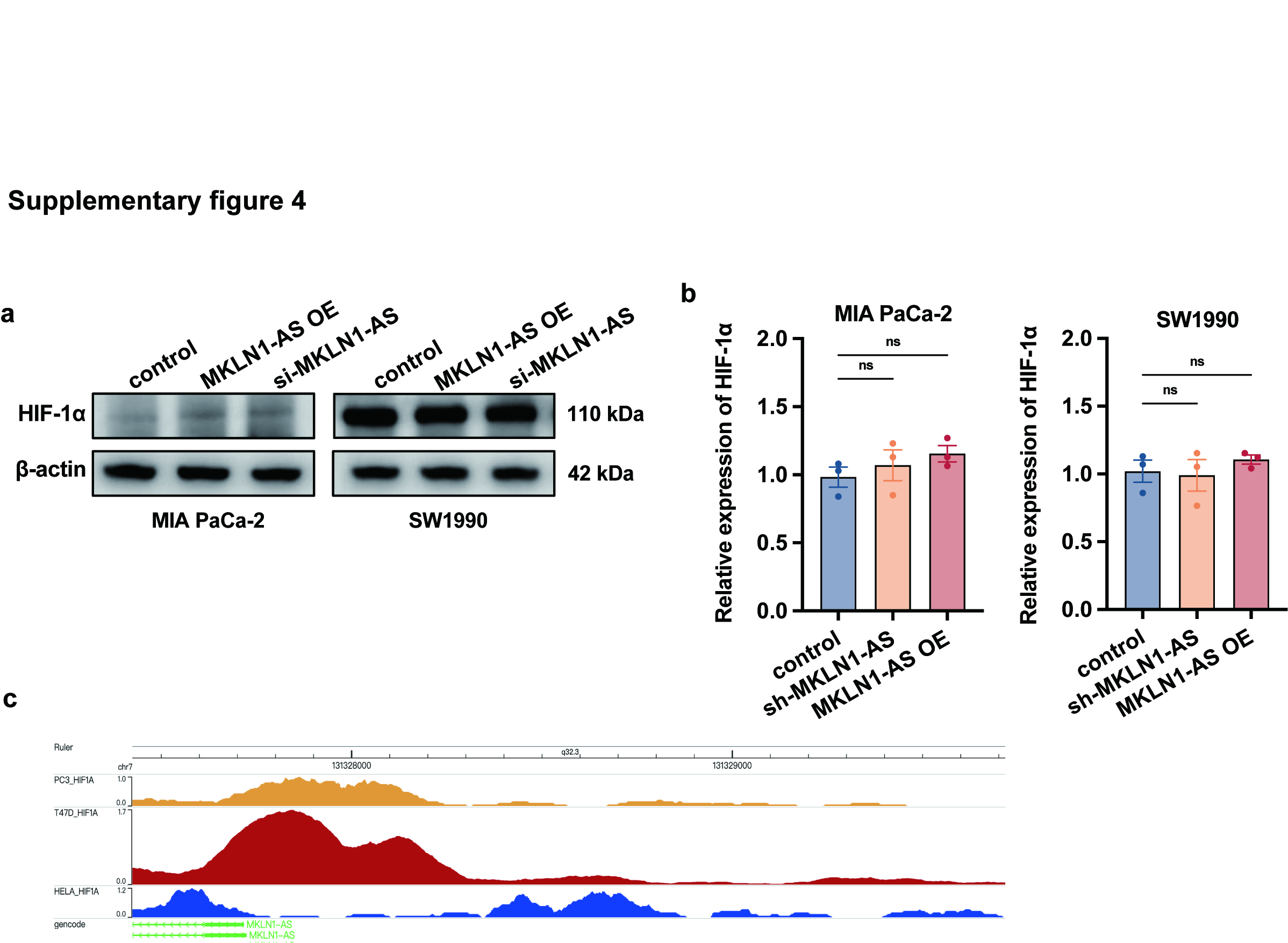

Supplement: Supplementary file 4 — Supplementary Figure 4. Transcriptionally regulation of MKLN1-AS expression in pancreatic cancer by HIF-1α. a, b Western-blot and RT-qPCR assay of the expression of HIF-1α with MKLN-AS overexpressed vector or MKLN1-AS knockdown siRNA. c CHIP-seq data obtained from the Cistrom database, HIF-1α ChIP-seq technique was employed to investigate the binding interactions between the promoter region of the MKLN1-AS and the HIF-1α in cancer cells. Data are means ± SEM and are representative of at least 3 independent experiments. (*P≤0.05, **P≤0.01, and ***P≤0.001. NS, not significant). (TIF 17645 kb) [file 10565_2024_9863_MOESM4_ESM.tif]
