# Supplementary material for: MKLN1-AS promotes pancreatic cancer progression as a crucial downstream mediator of HIF-1α through miR-185-5p/TEAD1 pathway
Source: Cell Biol Toxicol. 2024 May 13;40(1):30. doi: 10.1007/s10565-024-09863-8 (PMC11090931; doi:10.1007/s10565-024-09863-8)
Supplement: Supplementary file 5 — (DOCX 23 kb) [file 10565_2024_9863_MOESM5_ESM.docx]

Supplementary Table S1. Clinicopathologic characteristics of the CH cohort pancreatic cancer patients from whom the tumor specimens were obtained.

| **Clinicopathologic**  **characteristic** | **N** | **%** |
| --- | --- | --- |
| Median age (N=25) | 61 |  |
| *Sex (N=25)* |  | |
| Male | 13 | 52 |
| Female | 12 | 48 |
| *Tumor size (N=25)* |  | |
| $\leq4$cm | 9 | 36 |
| >4cm | 16 | 64 |
| *T category (N = 25)* |  |  |
| T2 | 8 | 32 |
| T3 | 3 | 12 |
| T4 | 14 | 56 |
| *N category (N=25)* |  |  |
| N0 | 5 | 20 |
| N1 | 16 | 64 |
| N2 | 4 | 16 |
| *M category (N=25)* |  |  |
| M0 | 15 | 60 |
| M1 | 10 | 40 |
| *TNM category (N=25)* |  |  |
| II | 8 | 32 |
| III-IV | 17 | 68 |

Supplementary Table S2. Clinicopathologic characteristics of the TCGA cohort pancreatic cancer patients from whom the tumor specimens were obtained.

| **Clinicopathologic**  **characteristic** | **N** | **%** |
| --- | --- | --- |
| Median age (N=171) | 64 |  |
| *Race (N=171)* |  |  |
| Asian | 11 | 6.4 |
| Black or African American | 6 | 3.5 |
| Others | 4 | 2.3 |
| White | 150 | 87.7 |
| *Sex (N=171)* |  |  |
| Male | 93 | 54.3 |
| Female | 78 | 45.6 |
| *Tumor size (N=158)* |  | |
| $\leq4$cm | 105 | 66.5 |
| >4cm | 53 | 33.5 |
| *TNM category (N=168)* |  |  |
| I | 19 | 11.3 |
| II | 142 | 84.5 |
| III-IV | 7 | 4.2 |

Supplementary Table S3. Clinicopathologic characteristics of the GEO cohort pancreatic cancer patients from whom the tumor specimens were obtained.

| **Clinicopathologic**  **characteristic** | **N** | **%** |
| --- | --- | --- |
| *Sex (N=63)* |  |  |
| Male | 33 | 52.4 |
| Female | 30 | 47.6 |
| *Histology grade (N=63)* |  | |
| Well-differentiated | 6 | 9.5 |
| Moderate-differentiated | 35 | 55.6 |
| poor-differentiated | 18 | 28.6 |
| NA | 4 | 6.3 |
| *TNM category (N=63)* |  |  |
| IB | 13 | 20.6 |
| IIA | 17 | 27 |
| IIB | 33 | 52.4 |

Supplementary Table S4. The gene-specific primers, siRNA, and shRNA sequences were used in real-time PCR analysis, vector constructs, and ChIP analysis.

| **Gene** | **Forward primer (5’-3’)** | **Reverse primer (5’-3’)** |
| --- | --- | --- |
| **Primers for real-time quantitative PCR** | | |
| AC073046. 1 | TCTGAGGGCTGCTGCTGGT | AAGTGGCTTCAGTCTCGTGGAT |
| EGOT | GCCATACCGACTGICCAACTA | ССАСССТСССТТАТТТСТТТТ |
| CCR5AS | CTACTGTCAGCAGGGAGGAAGGT | CGTATTGAATCAGGGGIGG |
| LINCO1094 | CGATTTAGGCACGCTACATTC | ACAGATGGATGGCATTACTTAGAC |
| HCP5 | GAATGGTCCTGCTTTGGTGTC | ATTTCCAAGAGCCCAGACCC |
| AC011352.3 | TCGCAGGCTATTGAGGCAG | CATTTAGTTGTGAATCCCCAGTG |
| MKLN1-AS | GAGTAAGTCAGCAGGATTCACAG | CTTCAACCCAAGAGTAGGAGG |
| AC004837.2 | TTCCAGAATCCTGCCACTT | GTGTCACAAGAGAAAAAGAACCAG |
| TNFRSF10A-AS1 | GTGGCTCACGCCCATAAT | AGTTCACACATACAACTTCTACCTG |
| ITGB1-DT | GGCAAATCGGGACCGTGAG | CAGGCAAGCACATTATCACTCCA |
| ITGB1-DT | GATGGCTCTGTAGCTGCAAGT | TTGGCAAACTCTGGTGGG |
| AC015712. 2 | GGCACCTCCACTTCCCTTCT | TCCTATGCGAAGTTGTGGCTAA |
| UCA1 | CTTAGTGGCTGAAGACTGATGC | CCATTGAGGCTGTAGAGTTTGA |
| NKILA | GGCACTGACCGCTTCTGTTT | GCCATCTGGGGTAGACGCT |
| PAXIP1-AS2 | CTCTTTCTTACCCATTCAAACCAG | GGTGGATACCTTGCGGAGC |
| AC004943.2 | GGAAAACTCCCTGCTAGAGAGACT | ACCGTTCAGGTAAATCGTTGG |
| AL157838. 1 | CCTGAGGAACAACTAAATCTGC | CCTTGGCAAACTCGGTCA |
| AL121603.2 | CCAGCATTCTTGAAACTAACG | TTCGCTAGTAAATCCATAATCCT |
| AC068580.3 | GCCCAATGTTTCTCCAACCG | GAGCAAGCAGATGCACGCA |
| HIF-1α | GAACGTCGAAAAGAAAAGTCTCG | CCTTATCAAGATGCGAACTCACA |
| TEAD1 | GAAAGGGCCCTCAAAATGCC | AACCTTGCATACTCCGTCTCT |
| β-actin | GAATTCATTTTTGAGACCTTCAA | CCGGATCCATCTCTGCCTCGAAGTCC |
| miR-185-5p | AAGCGGATGGAGAGAAAGGCAG | ATCCAGTGCAGGGTCCGAGG |
| miR-148b-3p | ATGTGCGTCAGTGCATCACAGA | ATCCAGTGCAGGGTCCGAGG |
| miR-185-5p RT primer：GTCGTATCCAGTGCAGGGTCCGAGGTATTCGCACTGGATACGACTCAGGA  miR-148b-3p RT primer：GTCGTATCCAGTGCAGGGTCCGAGGTATTCGCACTGGATACGACACAAAG | | |
| U6 | CTCGCTTCGGCAGCACA | AACGCTTCACGAATTTGCGT |
| **Primers for CHIP-PCR analyses** | | |
| HRE1 | GCCATCTTGTCAGCACCGTA | CGTGGGAAACCCTGAGATAGG |
| HRE2 | AGCAGCTGAACTAAGACTCCG | TTCCTCCACCTACCTTCCCG |
| HRE3 | GGACTGGGAAACGTGTCTGT | CAGGCGGGGAGAACTTGTAG |
| **Sequences of siRNA and shRNA for target genes** | | |
| si-HIF-1α | CACCTATGACCTGCTTGGTGCTGAT | |
| sh-HIF-1α | GCCGAGGAAGAACTATGAACA | |
| sh-MKLN1-AS | sh#1 GCCTGGACAGTGTCATCATCT  sh#2 GCATGAGCCTTTCTGGCAATG  sh#3 TGAACACTCTTTTTAGAAA | |
| si-TEAD1 | GCTTGAATCAGTGGACATTCG | |
