# Supplementary material for: MKLN1-AS promotes pancreatic cancer progression as a crucial downstream mediator of HIF-1α through miR-185-5p/TEAD1 pathway
Source: Cell Biol Toxicol. 2024 May 13;40(1):30. doi: 10.1007/s10565-024-09863-8 (PMC11090931; doi:10.1007/s10565-024-09863-8)
Supplement: Supplementary file 6 — (PDF 3205 kb) [file 10565_2024_9863_MOESM6_ESM.pdf]

20240317010759966545536596209664

WORD COUNT 6723

TIME SUBMITTED 17-MAR-2024 01:08AM

PAPER ID 107611943

MKLN1-AS promotes pancreatic cancer progression as a crucial downstream mediator of HIF-1α through miR-185-5p/TEAD1 pathway.

Abstract

In pancreatic ductal adenocarcinomas (PDAC), profound hypoxia plays key roles in regulating cancer cell behavior, including proliferation, migration, and resistance to therapies.The initial part of this research highlights the important role played by long noncoding RNA (lncRNA) MKLN1-AS, which is controlled by hypoxia-inducible factor-1 alpha (HIF-1α), in the progression of PDAC.Human samples of PDAC showed a notable increase in MKLN1-AS expression, which was linked to a worse outcome.Forced expression of MKLN1-AS greatly reduced the inhibitory impact on the growth and spread of PDAC cells caused by HIF-1α depletion.Experiments on mechanisms showed that HIF-1α influences the expression of MKLN1-AS by directly attaching to a hypoxia response element in the promoter region of MKLN1-AS.MKLN1-AS acts as a competitive endogenous RNA (ceRNA) by binding to miR-185-5p, resulting in the regulation of TEAD1 expression and promoting cell proliferation, migration, and tumor growth.TEAD1 subsequently enhances the development of PDAC.Our study results suggest that MKLN1-AS could serve as a promising target for treatment and a valuable indicator for predicting outcomes in PDAC. PDAC is associated with low oxygen levels, and the long non-coding RNA MKLN1-AS interacts with TEAD1 in this context.

Keywords: PDAC; hypoxia; lncRNA; MKLN1-AS; TEAD1

1. Introduction

Pancreatic cancer, holding the seventh position in cancer-related deaths, stands as one of the deadliest human cancers [1]. Pancreatic ductal adenocarcinoma (PDAC) is the most common type of pancreatic cancer, representing 90% of cases [2]. The poor prognosis of PDAC has been attributed to a variety of factors, including challenges in early detection and resistance to drugs [3,4]. Currently, available effective therapeutics against PDAC remain limited. Thus, the investigation of targeted molecular therapies is urgently needed to improve outcomes of PDAC.

The hypoxic tumor microenvironment is a prevalent characteristic of PDAC, that contributes to tumor progression and negatively impacts on prognosis[5]. Intratumoral necrosis results from uncontrolled tumor

proliferation and fibrosis formation, which surpass the available oxygen supply in PDAC [2]. Oxygen availability greatly influences the expression and activity of HIF-1α. Unlike normoxic environments, PHD-containing proteins quickly hydroxylate HIF-1α, causing it to be polyubiquitinated by the tumor suppressor pVHL [6]. This results in its accumulation in the nucleus during hypoxic conditions, leading to nuclear translocation. Nuclear HIF-1α forms a heterodimer with HIF-β, facilitating the binding to hypoxia-responsive elements (HREs) in the promoters and thereby promoting the transcription of hypoxia-responsive genes [7].

Long non-coding RNAs (lncRNAs) are RNA molecules that consist of more than 200 nucleotides. Multiple studies have shown that the abnormal expression of lncRNAs plays a role in various biological functions in cancer cells, such as growth, movement, metabolic processes, cell death, and resistance to drugs [8–11]. Hypoxia-induced activation of HIF-1α mediated mounting aspects of oncogenic mechanisms to regulate cancer development, especially lncRNA dysregulation. Hypoxia-responsive lncRNAs are transcribed under the regulation of HIF-1α. Previous studies have reported activation of lncRNAs STEAP3-AS1 and LUCAT1 by HIF-1α leads to promoted tumor progression [12,13]. However, therapeutic strategies targeting HIF-1α signaling have shown limited efficacy [14], targeting HIF-1α downstream effector molecules can be a potentially successful alternative approach in PDAC treatment.

The present research shows that a hypoxia-inducible lncRNA named MKLN1-AS is upregulated in PDAC tumor tissues. Increased expression of MKLN1-AS was associated with unfavorable outcomes in individuals with PDAC, aligning with earlier research indicating the involvement of MKLN1-AS in the advancement of hepatocellular carcinoma [15]. Our study aims to elucidate the biological functions of MKLN1-AS in PDAC, which is governed by the regulation of HIF-1α. Our findings indicated that elevated levels of MKLN1-AS in PDAC act as a ceRNA by interacting with miR-185-5p, resulting in enhanced cell proliferation, migration, and tumor development through the modulation of TEAD1 expression.

2. Materials and methods

2.1 Patients and tissue species

Twenty-five people diagnosed with PDAC at Changhai Hospital provided tumor tissues and adjacent non-cancerous pancreatic tissues for analysis. The participants in the study were granted written informed

consent. The features of the patients were recorded in Supplementary Table 1. The diagnosis of PDAC in all patients was conclusively confirmed through surgical procedures and pathological examinations. Before the surgical procedure, none of the patients had received radiotherapy or chemotherapy treatments. Upon receiving the tissue samples from patients, they were promptly frozen in liquid nitrogen and stored at -80℃.

2.2 Extraction of RNA and performing real-time PCR

RNA was extracted from tissue and cell cultures with TRIzol® reagent (Invitrogen, MA, USA). RNA concentrations and quality were assessed using UV spectrophotometry. RNAs were reverse transcribed using the HiScript® III 1st Strand cDNA Synthesis Kit from Vazyme in Nanjing, China, following the provided guidelines. The quantitative real-time PCR (qPCR) assay was conducted using the SYBR-green mastermix (Vazyme, Nanjing, China) and done on LightCycler 480II equipment (Roche, Mannheim, Germany).In both mRNA and lncRNA, endogenous control was utilized via b-actin. The qPCR temperature procedure includes starting with a denaturation step at 95℃ for 5 minutes, then proceeding with 40 cycles of temperature fluctuation between 95℃ and 65℃ for 45 seconds each.cDNA synthesis and qPCR were used to assess the expression levels of microR-185-5p and microR-148b-3p, with the miScript RT kit (Qiagen GmbH) and miScript SYBR Green PCR kit (Qiagen GmbH) being employed, respectively. PCR primers can be found in Supplementary Table 3.

2.3 Cell culture and treatment

Cell lines from the American Type Culture Collection, including PANC-1, AsPC-1, CaPAN-2, SW1990, BxPC-3, and MIA PaCa-2, were acquired for the study of HPNE and human PDAC. Cells were maintained in Dulbecco's modified Eagle's medium (DMEM) with 10% fetal bovine serum (Invitrogen, Carlsbad, CA, USA) and 1% penicillin/streptomycin. Cells were cultured at 37 °C in the atmosphere with 5% carbon dioxide.

In the hypoxic culture experiment, the cells were subjected to an incubation environment consisting of 94% nitrogen (N_2), 1% oxygen (O_2), and 5% carbon dioxide (CO_2). The treatment of 250μM cobalt chloride ($CoCl_2$) for 24 hours induced hypoxia.

Subcellular RNA extraction was performed according to the manufacturer's instructions using a PARISTM Kit (Ambion). Quantitative reverse transcription polymerase chain reaction (qRT-PCR) was

used to further analyze RNA in the cytoplasm and nucleus. β-actin and U6 served as controls for cytoplasmic and nuclear compartments, respectively.

2.4 Oligonucleotides and transfection

In Shanghai, China, GenePharma created vectors for overexpression, shRNA, and siRNA that target HIF-1α, MKLN1-AS, and TEAD1. A lentivirus vector carrying these sequences (GenePharma, Shanghai, China) was also constructed containing matching controls. MiR-185-5p mimics, miR-185-5p inhibitor, and their respective negative controls were supplied by GenePharma in Shanghai, China. PDAC cells were transfected with the oligonucleotides using Lipofectamine 3000 (Invitrogen, USA), followed by confirmation of transfection efficiency through qRT-PCR analysis.

2.5 Western blot analysis

The cell lysate was created by treating the cells with RIPA buffer from Epizyme in Shanghai, China, and the amount of protein was measured using the BCA Protein Quantification Kit. A 30 μg protein sample was subjected to electrophoretic separation on a 12% SDS-polyacrylamide gel. After electrophoresis, the proteins were moved to PVDF membranes. Following incubation with a 5% skim milk solution in TBS-T for four hours at room temperature, primary antibodies against TEAD1 (diluted 1:2000, purchased from Abcam, catalog number ab133533, UK) and HIF-1α (diluted 1:2000, acquired from Abcam, catalog number ab79546, UK) were left overnight at 4°C.To ensure equal loading of protein samples, an anti-β-actin antibody (diluted at 1:5000, sourced from Abcam, catalog number ab6275, United Kingdom) was employed. After washing the membranes three times with TBS-T for 15 minutes each, they were then incubated with secondary antibodies (diluted at 1:10,000; Westang) conjugated to horseradish peroxidase for 45 minutes at room temperature. Protein bands were visualized using a developing solution (Vazyme, Nanjing, China) and exposed to X-ray film.

2.6 Bioinformatic analysis

Data on clinical characteristics and gene expression in relation to PDAC were acquired from The Cancer Genome Atlas (TCGA) database and Gene Expression Omnibus (GEO) database, available at https://portal.gdc.cancer.gov/ and https://www.ncbi.nlm.nih.gov/geo/ respectively. All data included 171 PDAC patients from the TCGA database and 63 PDAC patients from the GEO database, and the quality controls were analyzed by R language. The details of data from the public database are exhibited in

Supplementary Tables 2, and 3. Levels of gene expression were standardized and then divided into groups based on a predetermined threshold to distinguish between high and low expression. Correlation maps involving two genes were generated using the ggstatsplot software in R, while heatmaps displaying correlations among multiple genes were visualized using the R software package. Molecular interactions and relevant clinical data were analyzed using the R programming language.

Shared microRNAs (miRNAs) between MKLN1-AS and TEAD1 were identified using the miRcode and TargetScan databases, available at http://www.mircode.org/index.php and http://www.targetscan.org/vert_72/, respectively. The downstream miRNA and binding site sequences of MKLN1-AS were obtained from the JEFFERSON database, accessible at https://cm.jefferson.edu/rna22/Interactive/ .

2.7 Cell counting kit-8 (CCK-8) assay.

Cell counting kit-8 (CCK-8) assay was performed to count cells. A controlled climate incubation environment at 37°C for 2 hours was used for incubation of cells in 96-well microtiter plates. Cells were placed in 96-well microtiter plates at a concentration of 2×10^3 cells per well and kept at 37°C for 2 hours in a regulated incubation setting. After a 24-hour incubation period, every well was treated with 10 microliters of the Cell Counting Kit-8 (CCK-8, Dojindo, Japan). Following incubation, the plates were washed twice with phosphate-buffered saline (PBS). Afterward, absorbance at 450 nm was measured using a microplate reader following the guidelines provided by the manufacturer.

2.8 Colony formation assay

Pancreatic cancer cells were placed in 6-well dishes with a concentration of 1×10^3 cells per well. Colonies with a cell count exceeding 200 cells per colony were subjected to a 10-day incubation period, followed by fixation for 15 minutes using a 4% paraformaldehyde solution. Afterward, the colonies were treated with a 0.1% crystal violet solution for a duration of 15 minutes. The number of colonies in each well was quantified using Image J analysis software (version 1.48), and the mean colony area (mm^2) was computed to facilitate comparisons of size. Three replicates of each experiment were conducted.

2.9 EdU assay

The experiment involving EdU (5-ethynyl-2'-deoxyuridine) proliferation was carried out following the guidelines provided by the manufacturer, utilizing the Cell-Light EdU Apollo 567 In Vitro Imaging Kit from Ribobio. Briefly, cells were processed according to the suggested procedures and then placed in 96-well plates at a density of around 5×10^3 cells per well. Each well received 100 microliters of medium containing 50 micromolar (μM) EdU, 24 hours after seeding. Afterward, the cells were placed in an incubator at a temperature of 37 degrees Celsius for a duration of 2 hours. After the cells were incubated, they were treated with a 4% paraformaldehyde solution and then stained with a mixture of Hoechst and Apollo reagents. Images were acquired using fluorescence microscopy and subsequently merged with Image J analysis software (version 1.48). Relative Edu incorporation was used for quantification analysis, followed by determining the total cell count and the count of EdU-positive cells in each field.

2.10 Cell migration/invasion assay

The test was performed in Boyden chambers with 24 wells, each holding 12 Millipore inserts. These inserts had an 8 mm pore size and were coated with a layer of basement membrane matrix on top, or left uncoated. Tests were performed in Boyden chambers using 24-well tissue culture plates, each equipped with 12 Millipore inserts. These inserts had a polycarbonate membrane with 8μm pores and were coated with either an ECMatrix layer (for invasion experiments) or left uncoated (for migration experiments). The bottom compartments were filled with a solution that included 10% fetal bovine serum, which acted as a chemoattractant.300 μL of serum-free medium contained 5 × 105 cells in the chambers above. Cells that infiltrated the ECMatrix or moved through the polycarbonate membrane were observed and documented under a microscope following 48 hours of incubation at 37°C.

2.11 Scratch wound healing assays

Pancreatic cancer cells were grown in 6-well dishes until they covered the surface, then manually scraped using a 20μl pipette tip. Next, a wound was induced by the addition of phosphate-buffered saline (PBS). A wound was created by manually scratching a cell monolayer with a 20-μl pipette tip, then rinsing with phosphate-buffered saline (PBS).Afterward, the cells were cultured for at least 12 hours in a medium containing 1% FBS, a level that does not impact the growth of pancreatic cancer cells. We established specific time points for capturing images within the cell growth cycle. Images of the wound healing process were captured using a phase contrast microscope. Image J analysis software (version 1.48) was utilized to measure the regions where cells migrated.

2.12 In vivo assay

Male BALB/C athymic nude mice, five weeks old, were maintained in an environment devoid of pathogens. The Animal Research Ethics Committee at Second Military Medical University approved the animal study in advance. To monitor tumor growth progression, we administered 0.1 ml of Hank's balanced salt solution into the flank of every mouse, utilizing 1×10^6 MIA PaCa-2 and SW1990 cells. Tumor volumes were measured weekly using the formula length × width^2 × 0.5. Following a period of four weeks, the mice were put to death, and the tumors were removed.An extra set of mice received injections of MIA PaCa-2 and SW1990 cells (1×10^6 each) via their ileocolic veins four weeks later for the metastasis test. All mice were euthanized on the 28th-day post-injection or upon reaching a pre-mortal state. Liver resection was performed, and the number of surface metastatic lesions on the livers was quantified.

2.13 Construction of MKLN1-AS promoter reporter plasmids

The MKLN1-AS segment, spanning from -891 to -4 in relation to the start of transcription, was inserted into a pGL3-basic vector (Promega), resulting in a 1000-bp DNA piece. This facilitated the generation of the reporter plasmid, designated as pGL3-891, which was purposefully engineered to encompass multiple hypoxia response elements (HREs). The nomenclature for the promoter-reporter plasmids was systematically based on the specific initiation site of the HREs. Subsequently, deletion mutation reporters were meticulously crafted for the plasmids, namely pGL3-681 and pGL3-30.To validate the integrity of these constructs, comprehensive sequencing was conducted on both the inserted sequences and the flanking regions of the plasmids.

2.14 RNA immunoprecipitation (RIP)

The RIP method was carried out using the Magna RIP™ RNA-Binding Protein Immunoprecipitation Kit from Millipore in Germany, following the instructions given, and abiding by the guidelines provided by the producer. Roughly 10 million SW1990 and MIA PaCa-2 cells were lysed using RIP lysis buffer. Following centrifugation at 10,000 times gravity and 4 degrees Celsius for 5 minutes, the PDAC cells were exposed to magnetic beads linked with human Ago2 antibodies obtained from Abcam in Cambridge, UK, or control IgG antibodies from Millipore in the USA. This overnight incubation occurred at 4°C.

Subsequently, the samples were treated with Proteinase K while agitating to facilitate protein digestion. The immunoprecipitated RNAs were then extracted and subjected to qRT-PCR analysis.

2.15 Chromatin immunoprecipitation assay

The chromatin immunoprecipitation assay kit (Millipore) was used to prepare 2×10^6 PDAC cells as specified in the manufacturer's instructions for the chromatin immunoprecipitation (ChIP) assay. DNA samples were first treated to induce precipitation before undergoing PCR amplification aimed at specific regions of the MKLN1-AS promoter. The resulting PCR products were then separated by electrophoresis on a 2% agarose gel and visualized with ethidium bromide staining.

2.16 Promoter construction and Dual-luciferase reporter assay

The promoter sequence of MKLN1-AS was identified using the UCSC gene browser, accessible at https://genome.ucsc.edu/. To determine the binding site sequence responsible for interacting with a transcription factor and facilitating MKLN1-AS transcription, a search was conducted on the JASPAR database (https://jaspar.genereg.net). Chip-seq data for transcription factors were acquired from Cistrome Data Browser at http://cistrome.org/db.

Recombinant luciferase plasmids were created by inserting the target fragments of MKLN1-AS or TEAD1 3'-UTR sequence, with wild-type (WT) or mutant-type (MT) miR-185-5p binding sites, into the pGL3-Basic luciferase vector from Promega in the USA. Transfection of cells was performed with the wild-type (WT) or mutant (MUT) luciferase reporter plasmid, along with miR-185-5p mimics, inhibitors, or negative control (NC), using Lipofectamine 3000 (Life Technologies Corporation, Carlsbad, CA, USA). After incubating for 48 hours, the Promega Dual-Luciferase assay was employed to measure the firefly and Renilla enzyme activity. Creating vectors for overexpressing or silencing the HIF-1α transcription factor, as well as wild-type or mutant MKLN1-AS sequences, is essential for conducting the combination test. The procedure remains unchanged. The relative luciferase activities were determined by subtracting the Renilla luciferase activities from the firefly luciferase activities. The analysis was conducted in triplicate for each group.

2.17 Statistical analysis

The mean ± SME of the data obtained from three triplicated independent experiments was reported. Data analysis was performed with the assistance of GraphPad Prism software (Intuitive programmer for

Science, San Diego, CA) and SPSS 26.0 (SPSS Inc., Chicago, IL, USA). The comparison between two normal distribution groups was conducted using the Two-tailed Student's t-test. The disparity between the two abnormal distribution groups was analyzed by nonparametric tests. For the analysis of numerous groups, a one-way ANOVA was undertaken. Survival curves were compared using log-rank tests and depicted using the Kaplan-Meier method. Genes were analyzed for correlation using Pearson's correlation coefficient. Statistical significance was determined by using P values below 0.05 in all conducted tests.

Result

3.1 Identification of MKLN1-AS as a key hypoxia-responsive lncRNA in PDAC carcinogenesis

To identify downstream effective lncRNAs to HIF-1α (i.e., hypoxia-responsive lncRNAs), we conducted an analysis of mRNA expression profiles from the TCGA database of PDAC. A three-step screening strategy was used: 1. lncRNAs showing a significant positive correlation with HIF-1α expression, requiring a p-value below 0.05 and a correlation coefficient (r) above 0.4; 2. lncRNAs that displayed a negative association with overall survival, with a p-value less than 0.01; 3. lncRNAs correlating positively with cancer staging, having a logFC above 0.5 and a p-value below 0.01. In total, 19 lncRNAs met these criteria and were thus designated as candidate hypoxia-responsive lncRNAs (Fig. 1a).

$CoCl_2$ is a well-established activator of HIF-1α [7,16]. We further treated two PDAC cell lines (MIA PaCa-2 and SW1990) with 250μM $CoCl_2$ for 24 hours. Confirmation of HIF-1α activation was achieved through qRT-PCR and western blot analysis (Supplementary Fig.1a, b). Only one lncRNA, named MKLN1-AS, was consistently upregulated in both cell lines (Fig. 1b). Additionally, AC004943.2 and AL121603.2 were upregulated in Mia PaCa-2, while UCA1 and AC005479.1 were upregulated in SW1990. To examine the direct regulation of HIF-1α on these lncRNAs, we established HIF-1α-overexpressed and -knockdown cell lines (MIA PaCa-2 and SW1990) respectively. The transfection efficiency was confirmed through qRT-PCR and western blot analysis (Supplementary Fig.1c, d). Expression of MKLN1-AS changed accordingly with the enforced or decreased levels of HIF-1α, with the other lncRNAs did not (Supplementary Fig.1e). Positive correlation between HIF-1α and MKLN1-AS was identified in a tissue cohort with 25 PDAC cases from Changhai hospital (Fig. 1c, P = 0.02, Supplementary Table 1). In addition, we examined whether MKLN1-AS expression could be induced by hypoxia by treating PDAC cells with $CoCl_2$. The levels of MKLN1-AS expression in MIA PaCa-2 and

SW1990 cells were elevated with increasing treatment doses, as shown in figure 1d. MKLN1-AS expression significantly rose in PDAC cells when MIA PaCa-2 and SW1990 were subjected to hypoxia (1% O2) for 48 hours (Fig. 1e). Collectively, these findings identified MKLN1-AS is a key hypoxia-responsive lncRNA that is significantly involved in HIF-1α-mediated PDAC carcinogenesis.

3.2 MKLN1-AS is highly expressed in PDAC and negatively associated with prognosis.

We conducted qRT-PCR to determine MKLN1-AS expression levels in 25 pairs of PDAC and para-cancerous samples (CH cohort). In comparison with para-cancerous tissues, PDAC tissues exhibited a significant upregulation of MKLN1-AS performed by in situ hybridization (ISH) and qPCR assay(Fig. 2a, 2b, $P<0.001$). In addition, increased levels of MKLN1-AS were associated with greater tumor size ($P<0.01$, Fig. 2c), poorer differentiation grades (Fig. 2d, $P<0.05$), and shorter overall survival (OS) (Fig. 2e, $P = 0.04$). We further delved into transcriptome sequencing data obtained from 171 PDAC patients in the TCGA database (Supplementary Table 2). Consistent with our results in CH cohort, expression of MKLN1-AS expression was elevated in larger tissue ($P<0.01$, Fig. 2f), advanced histological grade (Fig. 2g, $P<0.01$), and shorter survival (Fig. 2h, $P = 0.03$). These findings suggest that MKLN1-AS could potentially serve as a marker for PDAC and may play vital roles in pancreatic carcinogenesis.

3.3 MKLN1-AS promoted PDAC development in vitro and in vivo.

Levels of MKLN1-AS expression were subsequently analyzed in both normal (HPNE) and cancerous pancreatic duct epithelial cells. Elevated levels of MKLN1-AS were identified throughout all PDAC cell lines in comparison with HPNE(supplementary Fig. 2a). To examine the biofunctions of MKLN1-AS, MKLN1-AS-overexpressed and -knockdown cell lines were constructed in MIA PaCa-2 and SW1990 cell lines respectively, and transfection efficacy was validated by qRT-PCR (supplementary Fig. 2b, c). shMKLN1-AS#2 was selected for following experiments based on its superior knockdown efficiency. The experiments using Cell-Counting Kit-8 test, Colony Formation, and Edu assay revealed that enforced expression of MKLN1-AS promoted proliferation of MIA PaCa-2 while knocking down of MKLN1-AS did the opposite in SW1990 cells (Fig. 3a-d). Because increased MKLN1-AS expression correlated with advanced PDAC TNM staging (Fig. 2c, f), we further investigated the role of MKLN1-AS in PDAC cells migration and invasion. Wound healing and transwell assay showed that elevated MKLN1-AS increased, and attenuated MKLN1-AS decreased the migratory and invasion capabilities of PDAC cells (Fig. 3e-h), indicating that MKLN1-AS is potentially involved in PDAC metastasis.

Considering the observed MKLN1-AS enhancement cells proliferation, migration, and invasion in vitro by MKLN1-AS, we conducted an investigation to ascertain the tumorigenic potential of MKLN1-AS utilizing xenograft mouse models. For the purpose of assessing tumor growth, we introduced PDAC cells either overexpressiong or with knockdown of MKLN1-AS subcutaneously into 5-week-old mice. Our findings demonstrated the transfection of MKLN1-AS significantly promoted the subcutaneous growth of MIA PaCa-2, whereas the knockdown of MKLN1-AS elicited the converse effect on SW1990 (Fig. 3i-k). These data further substantiate the essential role of MKLN1-AS in the development and progression of PDAC.

3.4 MKLN1-AS mediated the promotive effects of HIF-1α on PDAC development.

As our work identified that MKLN1-AS as an important hypoxia-responsive lncRNA, we further tested if MKLN1-AS was involved in the promotive effects of HIF-1α on PDAC. As shown in Fig. 4a-c, overexpression of MKLN1-AS significantly attenuated the inhibitory effects on MIA PaCa-2 cells by HIF-1α knockdown. In parallel, deletion of MKLN1-AS abolished the effects of HIF-1α in promoting cell proliferation (Fig. 4a-d). Scratch wound (Fig. 4e, f) and transwell (Fig. 4g, h) assays corroborated the parallel role of MKLN1-AS on HIF-1α-induced pro-tumor biofunctions.

We further conducted *in vivo* experiments to explore the involvement of MKLN1-AS in mediating the promotive effects of HIF-1α on tumor growth and metastasis. Increased levels of MKLN1-AS significantly reduced the inhibitory effects of sh-HIF-1α on MIA PaCa-2 cells, whereas the absence of MKLN1-AS eliminated the stimulatory effects of HIF-1α on SW1990 tumor growth (Fig. 4i-k).

To assess the metastatic potential of these tumors, we intravenously inject tumor cells into mice through the ileocolic vein. Enhanced expression of MKLN1-AS significantly counteracted the suppressive effects of sh-HIF-1α on hepatic metastasis in MIA PaCa-2 cells (Fig. 4l-n). Consistently, deletion of MKLN1-AS nullified the effects of HIF-1α on promoting hepatic metastasis in SW1990 cells. These data collectively identify the involvement of MKLN1-AS in mediating the promotive effects of HIF-1α on PDAC development.

3.5 MKLN1-AS promoted PDAC progression via elevated TEAD1 expression.

To investigate the target genes and relevant signaling pathways regulated by MKLN1-AS, we compared the gene expression profiles of SW1990 cells transfected with shMKLN1-AS to those of the control

group to. Deletion of MKLN1-AS was validated by qRT-PCR. Our analysis revealed that 44 genes were significantly downregulated in SW1990 cells with shMKLN1-AS (log2 fold change <-1, q < 0.01). Of the 44 genes mentioned above, 36 genes displayed significant correlations with HIF-1α by collecting data from Genecards public database, and we selected the top 50% of these genes based on their relevance scores for further analysis (Fig. 5a). In subsequent investigations, we conducted co-expression studies for these 18 genes with HIF-1α using two datasets: TCGA, which included 171 PDAC patients, and GEO dataset (GSE57495), consisting of 63 PDAC patients. Remarkably, we found that TEAD1, TGFBR2, and WWTR1 consistently exhibited statistically significant correlations with HIF-1α expression in both datasets (as shown in Supplementary Fig. 3a and 3b).

TEAD1, a crucial element of the Hippo signaling pathway, is vital for regulating the biological activities of YAP[17,18]. We further explored if TEAD1 was potentially involved in MKLN1-AS mediated development of PDAC. The data we collected indicated a positive correlation between MKLN1-AS and TEAD1 in PDAC tissues from the CH cohort, with a correlation coefficient of 0.468 and a significance level below 0.05 (supplementary Fig. 3c). The data from TCGA cohort consistently identified the positive correlation between TEAD1 and MKLN1-AS ($R = 0.286$, $P<0.001$) (Supplementary Fig. 3d). Analysis of survival using TCGA data revealed a significant link between elevated TEAD1 expression and decreased overall survival (P=0.02)(supplementary Fig. 3e). MKLN1-AS was found to promote TEAD1 expression in PDAC cells by western blotting and qRT-PCR, whereas knocking down MKLN1-AS inhibited TEAD1 expression(Fig. 5b).

Next, we investigated the role of TEAD1 in the impacts of MKLN1-AS on tumor cell aggressiveness. As illustrated in Fig. 5c-f, knocking down TEAD1 in MIA PaCa-2 cells abrogated the effects of MKLN1-AS on promoting cell proliferation. On the contrary, overexpressing TEAD1 notably reversed the growth inhibition in SW1990 cells induced by the knockdown of MKLN1-AS. We further tested if TEAD1 was involved in the promotive effects of MKLN1-AS on PDAC migration and invasion. Our results showed that deletion of TEAD1 abrogate the effects of MKLN1-AS on promoting cell migration and invasion (Fig. 5g, i), whereas overexpression of TEAD1 significantly attenuated the inhibitory effects on MIA PaCa-2 cells by MKLN1-AS knockdown (Fig. 5h, j). These findings collectively identified that the promotive effects on PDAC by MKLN1-AS were at least partially dependent on TEAD1.

3.6 MKLN1-AS functions as a miRNA sponge for miR-185-5p to regulate the expression of TEAD1.

To determine the subcellular localization of MKLN1-AS, we utilized a cellular fractionation assay and then conducted qRT-PCR analysis. The results, as depicted in Fig. 6a, indicated that MKLN1-AS primarily resides in the cytosol of PDAC cells, with a minor fraction present in the nucleus. Recognizing that cytoplasmic lncRNAs often function as competing endogenous RNA(ceRNA) [19,20], we constructed a ceRNA network to establish a connection between MKLN1-AS and TEAD1 via miRNAs. Initially, we discovered miRNAs linked to the survival rate of individuals with PDAC by analyzing the TCGA group Subsequently, we employed TargetScan and RNA22 v.2.0 to identify the shared miRNAs that can potentially bind to both MKLN1-AS and TEAD1 3'-UTRs. As illustrated in Fig. 6b, two miRNAs, miR-148b-3p and miR-185-5p, remained in the ceRNA network. The binding sites between MKLN1-AS and both miRNAs are presented in Fig. 6c.

We further conducted Ago2-RIP assay, showing miR-185-5p overexpression was significantly enriched for MKLN1-AS pulldown by Ago2. In comparison to IgG RIP, the increase in AGO2 RIP samples was eightfold (Fig. 6d). Subsequently, we designed reporter constructs with wildtype (MKLN1-AS-WT) and mutant (MKLN1-AS-Mut) miR-185-5p binding regions wihtin the MKLN1-AS sequence (Fig. 6e). miR-185-5p mimics transfection led to a notable decrease in luciferase activity in MKLN1-AS-WT reporters, while no effect was observed in MKLN1-AS-Mut reporters (Fig. 6f). In parallel, luciferase reporters were constructed with both wild-type and altered binding sites in the 3'-UTR areas of TEAD1 (Fig. 6g). Transfection with miR-185-5p mimics significantly reduced luciferase activity in reporters containing TEAD1 3'UTR-WT, but not in those with mutations (Fig. 6h). Furthermore, the decreased expression of MKLN1-AS is caused by the increased expression of miR-185-5p, whereas inhibition of miR-185-5p results in the upregulation of MKLN1-AS (Fig. 6i).

Furthermore, we explored the inhibition of TEAD1 by miR-185-5p in PDAC cells. As anticipated, the increase of miR-185-5p led to a reduction in TEAD1 abundance at both the protein and mRNA levels, while specific miR-185-5p inhibitors elevated TEAD1 levels in terms of protein and mRNA (Fig. 6j).

3.7 Transcriptional regulation of MKLN1-AS expression in pancreatic cancer by HIF-1α.

Expression of MKLN1-AS changed accordingly with the enforced or decreased levels of HIF-1α (supplementary Fig. 1d). Additionally, overexpression and knockdown of MKLN1-AS have little effects on HIF-1α expression, indicating that regulation of MKLN1-AS by HIF-1α is unidirectional (Supplementary Fig. 4a, b). Bioinformatics analysis focused on potential hypoxia response elements

(HREs) (5'-CGTG-3') in the promoter region [7,21] of MKLN1-AS, indicating the existence of several potential HIF-1α binding locations (Supplementary Fig. 4e). Three primary HIF-1α-binding locations were discovered in the MKLN1-AS promoter area, located at -891 (HRE1), -613 (HRE2), and -30 (HRE3) base pairs from the beginning of MKLN1-AS transcription (Fig. 7a). To further validate these findings, two deletion mutants (MKLN1-AS-700, covering HRE2 and HRE3; MKLN1-AS-400, covering only HRE3) and a reporter encompassing the full-length MKLN1-AS promoter (MKLN1-AS-1009, encompassing all HREs) were constructed . These reporters, along with HIF-1α expression vectors, were introduced into 293 T cells. Removing the area containing HRE1 significantly reduced the promoter function of MKLN1-AS, which was stimulated by HIF-1α, as shown in the luciferase reporter assay findings.(Fig. 7b). As illustrated in Fig. 7c, heightened HIF-1α expression in MIA PaCa-2 and SW1990 cells significantly augmented MKLN1-AS promoter activity. Moreover, a ChIP assay verified that HIF-1α directly interacted with HRE1 in both MIA PaCa-2 and SW1990 cells (Fig. 7d, e). Collectively, these data indicate that the activation of MKLN1-AS is, at least in part, a consequence of HIF-1α regulation.

Discussion

Numerous studies have reported that hypoxia-induced abnormal expression of non-coding RNAs promotes neoplasm progress. However, the specific molecular mechanism of hypoxia-related genes responsible for tumor development remained limited. HIF-1α, the primary transcription factor for responding to low oxygen levels, plays a vital role in the development of tumors, participating in processes such as blood vessel formation, red blood cell production, glucose metabolism, and inflammation [6]. Recent studies have reported that HIF-1α responsible for lncRNA PVT1-mediated pancreatic cancer enhancing [22]. A different research study found that has reported that the activation of lncRNA SLC2A1 declined the expression of HIF-1α to inhibit osteoarthritis [23]. During the research, it was found that lncRNA MKLN1-AS was a molecule downstream of HIF-1α, being activated at the transcriptional level by HIF-1α. HIF-1α binds to HREs located in the MKLN1-AS promoter to promote PDAC progression. Several studies have reported that MKLN1-AS correlates with targeted drug sensitivity, and is implicated in hypoxia-responsive lncRNAs, that predict HCC outcomes. However, the role and underlying mechanism of MKLN1-AS in PDAC have not been thoroughly explored[24]. In our present study, we discovered MKLN1-AS as a hypoxia-responsive lncRNA, via MKLN1-AS/miR-185-5p/TEAD1 pathway. This finding indicated that MKLN1-AS may serve as an alternative molecule

cooperating with targeting HIF-1α therapy for PDAC.

Located on chromosome 7, MKLN1-AS is a lncRNA that, along with five other lncRNAs (AC139491.2, AC145207.5, AC099850.3, AL590705.3, and AL049840.5), was initially identified as a biomarker for predicting the prognosis and response to immunotherapy in patients with Hepatocellular carcinoma HCC[25]. Furthermore, MKLN1-AS played a promoted oncogenic role in HCC induced by ferroptosis and predicted the outcomes of patients on immunotherapy [26]. This research unveiled a notable increase in MKLN1-AS expression in PDAC tissues when compared to non-cancerous samples, marking the first time this has been observed. PDAC patients with higher levels of MKLN1-AS have a poorer prognosis and low differentiation grades according to clinical data. And we discovered that enforced MKLN1-AS promoted PDAC cells proliferation and metastasis in vitro and in vivo. Further research exhibited that the function of MKLN1-AS promoted PDAC development via TEAD1 upregulated. Our research is the initial documentation pinpointing MKLN1-AS as a long non-coding RNA that responds to low oxygen levels in the development of PDAC, while also explaining the process by which MKLN1-AS contributes to the progression of PDAC.

An integral component of the Hippo signaling pathway, TEAD1 is known for its involvement in processes such as tumor cell proliferation, migration, epithelial-mesenchymal transition [27], and drug resistance [28]. The first report TEAD1 in PDAC promotion was acting as the transcription enhancer factor bound by MSLN element to enhance MSLN expression [29]. Following that, TEAD1 causes an elevation in 5-hydroxymethylcytosine levels, leading to alterations that allow for the identification of PDAC in its early stages [30]. Also, in this context, mounting research explored suppressors that inhibit the expression of TEAD1. Including short isoform (PRLR-SF) [31], Cyclin-Dependent Kinase 1 (CDK1) [32], and Fascin protein [33] contribute to suppress cell growth and spread by reducing TEAD1 expression. However, the mechanism of upregulated TEAD1 in pancreatic cancer is referred to less frequently. Our research demonstrated the increased expression of TEAD1 regulated by MKLN1-AS and validated the role of MKLN1-AS as a competing endogenous RNA (ceRNA) that interacts with miR-185-5p to upregulate TEAD1. In our molecular experiment, after inhibiting MKLN1-AS, the expression of TEAD1 decreased. This finding indicated that inhibiting MKLN1-AS disrupted the TEAD1 signaling pathway, offering a novel means for targeting inhibition of TEAD1 in PDAC.

Since MKLN1-AS primarily localized in the cytoplasm, we subsequently explored its potential role as a

competitive endogenous RNA (ceRNA), sequestering specific miRNAs targeting TEAD1. Our bioinformatic analysis and experiment evidence revealed miR-185-5p may be absorbed by MKLN1-AS, influencing the expression of TEAD significantly. Previous research has shown different results on the influence of miR-185-5p on cancer advancement, with contradictory functions observed as both inhibiting and promoting tumor growth [34-36]. A recent research discovered that miR-185-5p delivered through extracellular vesicles in the blood of individuals can serve as a marker for advanced adenoma and colorectal cancer [37]. In the field of PDAC research, it has been highlighted that miR-185-5p exerts regulatory control over non-protein coding RNA, thereby fostering the progression of pancreatic cancer [38]. The study unveils a novel suppressive function of miR-185-5p targeting the ondogene TEAD1, suggesting its potential utility as a biomarker for predicting PDAC prognosis.

To sum up, our investigation has elucidated the upregulation of a hypoxia-responsive lncRNA known as MKLN1-AS within pancreatic cancer tissue. This lncRNA functions as an oncogenic factor, contributing to the promotion of both tumor growth and metastasis. The elevated MKLN1-AS competitively interacts with miR-185-5p, resulting in TEAD1 expression increase. This molecular cascade contributes to the aggressiveness of PDAC. Targeting the HIF-1α/MKLN1-AS/miR-185-5p/TEAD1 signaling pathway holds promise as a future treatment strategy for PDAC.

Reference

1. Siegel RL, Miller KD, Fuchs HE, Jemal A. Cancer statistics, 2022. *CA Cancer J Clin*. 2022;72(1):7-33. doi:10.3322/caac.21708
2. Park W, Chawla A, O'Reilly EM. Pancreatic Cancer: A Review. *JAMA*. 2021;326(9):851-862. doi:10.1001/jama.2021.13027
3. Connor AA, Gallinger S. Pancreatic cancer evolution and heterogeneity: integrating omics and clinical data. *Nat Rev Cancer*. 2022;22(3):131-142. doi:10.1038/s41568-021-00418-1
4. Cai J, Chen H, Lu M, et al. Advances in the epidemiology of pancreatic cancer: Trends, risk factors, screening, and prognosis. *Cancer Lett*. 2021;520:1-11. doi:10.1016/j.canlet.2021.06.027
5. Tao J, Yang G, Zhou W, et al. Targeting hypoxic tumor microenvironment in pancreatic cancer. *J Hematol Oncol*. 2021;14(1):14. doi:10.1186/s13045-020-01030-w
6. Semenza GL. HIF-1 and mechanisms of hypoxia sensing. *Curr Opin Cell Biol*. 2001;13(2):167-171. doi:10.1016/s0955-0674(00)00194-0
7. Kong F, Kong X, Du Y, et al. STK33 Promotes Growth and Progression of Pancreatic Cancer as a Critical Downstream Mediator of HIF1α. *Cancer Res*. 2017;77(24):6851-6862. doi:10.1158/0008-5472.CAN-17-0067
8. Tan YT, Lin JF, Li T, Li JJ, Xu RH, Ju HQ. LncRNA-mediated posttranslational modifications and reprogramming of energy metabolism in cancer. *Cancer Commun (Lond)*. 2021;41(2):109-120. doi:10.1002/cac2.12108

9. Liu W, Tang J, Zhang H, et al. A novel lncRNA PTTG3P/miR-132/212-3p/FoxM1 feedback loop facilitates tumorigenesis and metastasis of pancreatic cancer. *Cell Death Discov*. 2020;6(1):136. doi:10.1038/s41420-020-00360-5

10. Zhao X, Su L, He X, Zhao B, Miao J. Long noncoding RNA CA7-4 promotes autophagy and apoptosis via sponging MIR877-3P and MIR5680 in high glucose-induced vascular endothelial cells. *Autophagy*. 2020;16(1):70-85. doi:10.1080/15548627.2019.1598750

11. Chen Q, Wang W, Wu Z, et al. Over-expression of lncRNA TMEM161B-AS1 promotes the malignant biological behavior of glioma cells and the resistance to temozolomide via up-regulating the expression of multiple ferroptosis-related genes by sponging hsa-miR-27a-3p. *Cell Death Discov*. 2021;7(1):311. doi:10.1038/s41420-021-00709-4

12. Huan L, Guo T, Wu Y, et al. Hypoxia induced LUCAT1/PTBP1 axis modulates cancer cell viability and chemotherapy response. *Mol Cancer*. 2020;19(1):11. doi:10.1186/s12943-019-1122-z

13. Zhou L, Jiang J, Huang Z, et al. Hypoxia-induced lncRNA STEAP3-AS1 activates Wnt/β-catenin signaling to promote colorectal cancer progression by preventing m6A-mediated degradation of STEAP3 mRNA. *Mol Cancer*. 2022;21(1):168. doi:10.1186/s12943-022-01638-1

14. Lee KE, Spata M, Bayne LJ, et al. Hif1a Deletion Reveals Pro-Neoplastic Function of B Cells in Pancreatic Neoplasia. *Cancer Discov*. 2016;6(3):256-269. doi:10.1158/2159-8290.CD-15-0822

15. Gao W, Chen X, Chi W, Xue M. Long non-coding RNA MKLN1-AS aggravates hepatocellular carcinoma progression by functioning as a molecular sponge for miR-654-3p, thereby promoting hepatoma-derived growth factor expression. *Int J Mol Med*. 2020;46(5):1743-1754. doi:10.3892/ijmm.2020.4722

16. Lv XM, Li MD, Cheng S, et al. Neotuberostemonine inhibits the differentiation of lung fibroblasts into myofibroblasts in mice by regulating HIF-1α signaling. *Acta Pharmacol Sin*. 2018;39(9):1501-1512. doi:10.1038/aps.2017.202

17. Sun T, Peng H, Mao W, et al. Autophagy-mediated negative feedback attenuates the oncogenic activity of YAP in pancreatic cancer. *Int J Biol Sci*. 2021;17(13):3634-3645. doi:10.7150/ijbs.61795

18. Zhao B, Ye X, Yu J, et al. TEAD mediates YAP-dependent gene induction and growth control. *Genes Dev*. 2008;22(14):1962-1971. doi:10.1101/gad.1664408

19. Fan H, Lv P, Mu T, et al. LncRNA n335586/miR-924/CKMT1A axis contributes to cell migration and invasion in hepatocellular carcinoma cells. *Cancer Lett*. 2018;429:89-99. doi:10.1016/j.canlet.2018.05.010

20. Xu YH, Deng JL, Wang G, Zhu YS. Long non-coding RNAs in prostate cancer: Functional roles and clinical implications. *Cancer Lett*. 2019;464:37-55. doi:10.1016/j.canlet.2019.08.010

21. Zhao X, Gao S, Ren H, et al. Hypoxia-inducible factor-1 promotes pancreatic ductal adenocarcinoma invasion and metastasis by activating transcription of the actin-bundling protein fascin. *Cancer Res*. 2014;74(9):2455-2464. doi:10.1158/0008-5472.CAN-13-3009

22. Sun J, Zhang P, Yin T, Zhang F, Wang W. Upregulation of LncRNA PVT1 Facilitates Pancreatic Ductal Adenocarcinoma Cell Progression and Glycolysis by Regulating MiR-519d-3p and HIF-1A. *J Cancer*. 2020;11(9):2572-2579. doi:10.7150/jca.37959

23. Guan Z, Jin X, Guan Z, Liu S, Tao K, Luo L. The gut microbiota metabolite capsiate regulate SLC2A1 expression by targeting HIF-1α to inhibit knee osteoarthritis-induced ferroptosis. *Aging Cell*. 2023;22(6):e13807. doi:10.1111/acel.13807

24. Tang P, Qu W, Wang T, et al. Identifying a Hypoxia-Related Long Non-Coding RNAs Signature to Improve the Prediction of Prognosis and Immunotherapy Response in Hepatocellular Carcinoma.

Front Genet. 2021;12:785185. doi:10.3389/fgene.2021.785185

25. Cheng Z, Han J, Jiang F, Chen W, Ma X. Prognostic pyroptosis-related lncRNA signature predicts the efficacy of immunotherapy in hepatocellular carcinoma. *Biochem Biophys Rep*. 2022;32:101389. doi:10.1016/j.bbrep.2022.101389

26. Fang C, Liu S, Feng K, et al. Ferroptosis-related lncRNA signature predicts the prognosis and immune microenvironment of hepatocellular carcinoma. *Sci Rep*. 2022;12(1):6642. doi:10.1038/s41598-022-10508-1

27. Li F, Negi V, Yang P, et al. TEAD1 regulates cell proliferation through a pocket-independent transcription repression mechanism. *Nucleic Acids Res*. 2022;50(22):12723-12738. doi:10.1093/nar/gkac1063

28. Wei L, Ma X, Hou Y, et al. Verteporfin reverses progestin resistance through YAP/TAZ-PI3K-Akt pathway in endometrial carcinoma. *Cell Death Discov*. 2023;9(1):30. doi:10.1038/s41420-023-01319-y

29. Hucl T, Brody JR, Gallmeier E, Iacobuzio-Donahue CA, Farrance IK, Kern SE. High Cancer-Specific Expression of Mesothelin (*MSLN*) Is Attributable to an Upstream Enhancer Containing a Transcription Enhancer Factor–Dependent MCAT Motif. *Cancer Research*. 2007;67(19):9055-9065. doi:10.1158/0008-5472.CAN-07-0474

30. Guler GD, Ning Y, Ku CJ, et al. Detection of early stage pancreatic cancer using 5-hydroxymethylcytosine signatures in circulating cell free DNA. *Nat Commun*. 2020;11(1):5270. doi:10.1038/s41467-020-18965-w

31. Nie H, Huang PQ, Jiang SH, et al. The short isoform of PRLR suppresses the pentose phosphate pathway and nucleotide synthesis through the NEK9-Hippo axis in pancreatic cancer. *Theranostics*. 2021;11(8):3898-3915. doi:10.7150/thno.51712

32. Zeng Y, Stauffer S, Zhou J, Chen X, Chen Y, Dong J. Cyclin-dependent kinase 1 (CDK1)-mediated mitotic phosphorylation of the transcriptional co-repressor Vgll4 inhibits its tumor-suppressing activity. *J Biol Chem*. 2017;292(36):15028-15038. doi:10.1074/jbc.M117.796284

33. Lin S, Li Y, Wang D, et al. Fascin promotes lung cancer growth and metastasis by enhancing glycolysis and PFKFB3 expression. *Cancer Lett*. 2021;518:230-242. doi:10.1016/j.canlet.2021.07.025

34. Scognamiglio I, Cocca L, Puoti I, et al. Erratum: Exosomal microRNAs synergistically trigger stromal fibroblasts in breast cancer. *Mol Ther Nucleic Acids*. 2022;29:656. doi:10.1016/j.omtn.2022.08.023

35. Değerli E, Torun V, Cansaran-Duman D. miR-185-5p response to usnic acid suppresses proliferation and regulating apoptosis in breast cancer cell by targeting Bcl2. *Biol Res*. 2020;53(1):19. doi:10.1186/s40659-020-00285-4

36. Yu M, Shi C, Xu D, et al. LncRNA ASB16-AS1 drives proliferation, migration, and invasion of colorectal cancer cells through re. :12.

37. Shi YJ, Fang YX, Tian TG, et al. Discovery of extracellular vesicle-delivered miR-185-5p in the plasma of patients as an indicator for advanced adenoma and colorectal cancer. *J Transl Med*. 2023;21(1):421. doi:10.1186/s12967-023-04249-6

38. Li H, Shen H, Xie P, et al. Role of long intergenic non-protein coding RNA 00152 in pancreatic cancer glycolysis via the manipulation of the microRNA-185-5p/Krüppel-like factor 7 axis. *J Cancer*. 2021;12(21):6330-6343. doi:10.7150/jca.63128

20240317010759966545536596209664

ORIGINALITY REPORT

21%
SIMILARITY INDEX

PRIMARY SOURCES

1	www.researchsquare.com<br>Internet	93 words	1%
2	www.nature.com<br>Internet	87 words	1%
3	www.spandidos-publications.com<br>Internet	53 words	1%
4	link.springer.com<br>Internet	47 words	1%
5	www.bio-bigdata.net<br>Internet	41 words	1%
6	www.mdpi.com<br>Internet	36 words	1%
7	www.frontiersin.org<br>Internet	32 words	1%
8	worldwidescience.org<br>Internet	29 words	< 1%
9	www.ncbi.nlm.nih.gov<br>Internet	29 words	< 1%
10	mdpi-res.com<br>Internet		

28 words — < 1%

11 healthdocbox.com
Internet
27 words — < 1%

12 www.dovepress.com
Internet
26 words — < 1%

13 www.scirp.org
Internet
26 words — < 1%

14 Fan Zhang, Zhiwei Wu, Bowen Yu, Zhengping Ning et al. "ATP13A2 activates the pentose phosphate pathway to promote colorectal cancer growth though TFEB-PGD axis", Clinical and Translational Medicine, 2023
Crossref
20 words — < 1%

15 Yongchang Guan, Wenjin Yang, Feng Zhang, Liming Zhang, Liang Wang. "CircPOSTN competes with KIF1B for miR-185–5p binding sites to promote the tumorigenesis of glioma", Brain Research Bulletin, 2022
Crossref
19 words — < 1%

16 Fazheng Shen, Haigang Chang, Guojun Gao, Bin Zhang, Xiangsheng Li, Baozhe Jin. "Long noncoding RNA FOXD2-AS1 promotes glioma malignancy and tumorigenesis via targeting miR-185-5p/CCND2 axis", Journal of Cellular Biochemistry, 2018
Crossref
18 words — < 1%

17 Hui Yang, Hanyu Zhou, Minjie Fu, Hao Xu, Haoyu Huang, Min Zhong, Mengying Zhang, Wei Hua, Kun Lv, Guoping Zhu. "TMEM64 aggravates the malignant phenotype of glioma by activating the Wnt/β-catenin signaling
18 words — < 1%

pathway", International Journal of Biological Macromolecules, 2024
Crossref

18 pure.uva.nl
Internet
18 words — < 1%

19 Ben George, Matthew Kent, Andy Surinach, Neil Lamarre, Paul Cockrum. "The Association of Real-World CA 19-9 Level Monitoring Patterns and Clinical Outcomes Among Patients With Metastatic Pancreatic Ductal Adenocarcinoma", Frontiers in Oncology, 2021
Crossref
17 words — < 1%

20 www.freepatentsonline.com
Internet
17 words — < 1%

21 Jiali Wang, Yuanyuan li, Fangfang Cao, Pei Yang. "Adipocytes play an etiological role in renal tubular epithelial cells of db/db mice", Research Square Platform LLC, 2023
Crossref Posted Content
16 words — < 1%

22 www.wjgnet.com
Internet
16 words — < 1%

23 "Role of Transcription Factors in Gastrointestinal Malignancies", Springer Nature, 2017
Crossref
15 words — < 1%

24 He Ren, Lingling Jia, Tiansuo Zhao, Huan Zhang, Jing Chen, Shaoguang Yang, Jingcheng Liu, Ming Yu, Jihui Hao. "Hypoxia inducible factor (HIF)-1α directly activates leptin receptor (Ob-R) in pancreatic cancer cells", Cancer Letters, 2014
Crossref
15 words — < 1%

25 Yiping Zhu, Fang Wu, Weiwei Gui, Nan Zhang, Erik Matro, Linghua Zhu, Daniel Turunen Eserberg, Xihua Lin. "A positive feedback regulatory loop involving the lncRNA PVT1 and HIF-1α in pancreatic cancer", Journal of Molecular Cell Biology, 2021 — 15 words — < 1%
Crossref

26 Chen Luo, Kang Lin, Cegui Hu, Xiaojian Zhu, Jinfeng Zhu, Zhengming Zhu. "LINC01094 promotes pancreatic cancer progression by sponging miR-577 to regulate LIN28B expression and the PI3K/AKT pathway", Molecular Therapy - Nucleic Acids, 2021 — 14 words — < 1%
Crossref

27 Kui-Xiang Wang, Li-Li Zhao, Ling-Tao Zheng, Li-Bin Meng, Liang Jin, Long-Jun Zhang, Fan-Lei Kong, Fang Liang. "Accelerated Wound Healing in Diabetic Rat by miRNA-185-5p and Its Anti-Inflammatory Activity", Diabetes, Metabolic Syndrome and Obesity, 2023 — 14 words — < 1%
Crossref

28 Wongpattaraworakul, Wattawan. "Novel Prognostic Biomarkers for Oral Squamous Cell Carcinoma", The University of Iowa, 2023 — 14 words — < 1%
ProQuest

29 X Cao. "Upregulation of VEGF-A and CD24 gene expression by the tGLI1 transcription factor contributes to the aggressive behavior of breast cancer cells", Oncogene, 01/05/2012 — 14 words — < 1%
Crossref

30 academic.oup.com — 14 words — < 1%
Internet

31 Honglong Wei, Zongzhen Xu, Feng Liu, Fuhai Wang, Xin Wang, Xueying Sun, Jie Li. "Hypoxia — 13 words — < 1%

induces oncogene yes-associated protein 1 nuclear translocation to promote pancreatic ductal adenocarcinoma invasion via epithelial–mesenchymal transition", Tumor Biology, 2017
Crossref

---

32 Qiuyan Zhao, Zhonglin Zhu, Wenqin Xiao, Guanzhao Zong et al. "Hypoxia-induced circRNF13 promotes the progression and glycolysis of pancreatic cancer", Experimental & Molecular Medicine, 2022
Crossref
13 words — < 1%

---

33 Wenyu Liu, Jian Tang, Huiqing Zhang, Fanyang Kong, Huiyun Zhu, Ping Li, Zhaoshen Li, Xiangyu Kong, Kaixuan Wang. "A novel lncRNA PTTG3P/miR-132/212-3p/FoxM1 feedback loop facilitates tumorigenesis and metastasis of pancreatic cancer", Cell Death Discovery, 2020
Crossref
13 words — < 1%

---

34 tcr.amegroups.com
Internet
13 words — < 1%

---

35 Fye, Lau Beng. "Cytotoxic and Apoptosis-Inducing Activities of the Extracts and Chemical Constituents from the Tiger's Milk Mushroom, Lignosus Rhinocerotis (Cooke) Ryvarden", University of Malaya (Malaysia), 2023
ProQuest
12 words — < 1%

---

36 nih.brage.unit.no
Internet
12 words — < 1%

---

37 www.sciencegate.app
Internet
12 words — < 1%

---

38 Lian Tang, Yiran Yin, Juncai Liu, Zhong Li, Xiaobo Lu. "MiR-124 Attenuates Osteoclastogenic
11 words — < 1%

Differentiation of Bone Marrow Monocytes Via Targeting Rab27a", Cellular Physiology and Biochemistry, 2017
Crossref

39 cancerres.aacrjournals.org 11 words — < 1%
Internet

40 cardiothoracicsurgery.biomedcentral.com 11 words — < 1%
Internet

41 core.ac.uk 11 words — < 1%
Internet

42 downloads.hindawi.com 11 words — < 1%
Internet

43 pubs.rsc.org 11 words — < 1%
Internet

44 www.iosrjournals.org 11 words — < 1%
Internet

45 Christopher L. Hunter. "Minocycline protects basal forebrain cholinergic neurons from mu p75-saporin immunotoxic lesioning", European Journal of Neuroscience, 6/2004 10 words — < 1%
Crossref

46 Qiyan Bao, Xiangling Liao, Rongyin Li, Nan Ding. "KCNQ1OT1 promotes migration and inhibits apoptosis by modulating miR-185-5p/Rab14 axis in oral squamous cell carcinoma", Development, Growth & Differentiation, 2019 10 words — < 1%
Crossref

47 Xiao-Jing Luo, Ming-Ming He, Jia Liu, Jia-Bo Zheng et al. "LncRNA TMPO-AS1 promotes esophageal 10 words — < 1%

squamous cell carcinoma progression by forming biomolecular condensates with FUS and p300 to regulate TMPO transcription", Experimental & Molecular Medicine, 2022
Crossref

48 aacrjournals.org — 10 words — < 1%
Internet

49 Akihisa Kato, Serina Ng, Amalraj Thangasamy, Haiyong Han et al. "A potential signaling axis between RON kinase receptor and hypoxia-inducible factor-1 alpha in pancreatic cancer", Molecular Carcinogenesis, 2021 — 9 words — < 1%
Crossref

50 Bhalchandra Mirlekar, Daniel Michaud, Ryan Searcy, Kevin Greene, Yuliya Pylayeva-Gupta. "IL-35 hinders endogenous anti-tumor T cell immunity and responsiveness to immunotherapy in pancreatic cancer", Cancer Immunology Research, 2018 — 9 words — < 1%
Crossref

51 He Zhu, Hongwei Zhang, Youliang Pei, Zhibin Liao et al. "Long non-coding RNA CCDC183-AS1 acts AS a miR-589-5p sponge to promote the progression of hepatocellular carcinoma through regulating SKP1 expression", Journal of Experimental & Clinical Cancer Research, 2021 — 9 words — < 1%
Crossref

52 Jiasheng Liu, Jie Zhu, Zhe Xiao, Xufeng Wang, Jianfei Luo. " contributes to colorectal cancer progression by sponging hsa- and targeting ", FEBS Open Bio, 2022 — 9 words — < 1%
Crossref

53 Landon-Brace, Natalie Catherine. "Investigating the Effect of Microenvironmental Gradients on — 9 words — < 1%

Tumour Cell Heterogeneity Using a 3D In Vitro Model of Pancreatic Cancer", University of Toronto (Canada), 2023
ProQuest

54 Weina Wang, Xiang Li, Canghai Guan, Zengtao Hu, Yuqiao Zhao, Wenzhi Li, Xingming Jiang. "LncRNA PCAT6 promotes the proliferation, migration and invasion of pancreatic ductal adenocarcinoma via regulating miR-185-5p/CBX2 axis", Pathology - Research and Practice, 2020 — 9 words — < 1%
Crossref

55 Zheli Niu, Guangwei Ren, Lijing Huang, Liqin Mu. "Circ_0008529 Contributes to Renal Tubular Cell Dysfunction in High Glucose Stress via miR-185-5p/SMAD2 Pathway in Diabetic Nephropathy", Biochemical Genetics, 2022 — 9 words — < 1%
Crossref

56 biosignaling.biomedcentral.com — 9 words — < 1%
Internet

57 ediss.uni-goettingen.de — 9 words — < 1%
Internet

58 jnanobiotechnology.biomedcentral.com — 9 words — < 1%
Internet

59 Airu Huang, Ling Ji, Yilong Huang, Qian Yu, Yufeng Li. "miR-185-5p alleviates CCI-induced neuropathic pain by repressing NLRP3 inflammasome through dual targeting MyD88 and CXCR4", International Immunopharmacology, 2022 — 8 words — < 1%
Crossref

60 Fanyang Kong, Xuan Deng, Xiangyu Kong, Yiqi Du et al. "ZFPM2-AS1, a novel lncRNA, attenuates the p53 pathway and promotes gastric carcinogenesis by stabilizing MIF", Oncogene, 2018 — 8 words — < 1%
Crossref

61	Jia Liu, Ze-Xian Liu, Jia-Jun Li, Zhao-Lei Zeng et al. " The Macrophage-Associated LncRNA Facilitates ILF3 Liquid–Liquid Phase Separation to Promote HIF1α Signaling in Esophageal Cancer ", Cancer Research, 2023<br>Crossref	8 words — < 1%
62	Min Wang, Mei-yuan Chen, Xing-jun Guo, Jian-xin Jiang. "Expression and significance of HIF-1α and HIF-2α in pancreatic cancer", Journal of Huazhong University of Science and Technology [Medical Sciences], 2015<br>Crossref	8 words — < 1%
63	Qian, Chen. "The Discovery and Pharmacological Elucidation of Novel Venom Neuropeptides From Two Species of Corals", University of Macau, 2023<br>ProQuest	8 words — < 1%
64	Xijun Chen, Qing Ye, Zhigao Chen, Qian Lin, Wen Chen, Chengrong Xie, Xiaomin Wang. "Long non-coding RNA muskelin 1 antisense RNA as a potential therapeutic target in hepatocellular carcinoma treatment", Bioengineered, 2022<br>Crossref	8 words — < 1%
65	Yuan Wu, Yu Jiang, Qiang Liu, Cui-Zhong Liu. "lncRNA H19 promotes matrix mineralization through up-regulating IGF1 by sponging miR-185-5p in osteoblasts", BMC Molecular and Cell Biology, 2019<br>Crossref	8 words — < 1%
66	diposit.ub.edu<br>Internet	8 words — < 1%
67	elibrary.tucl.edu.np<br>Internet	8 words — < 1%
	journals.plos.org	

68	Internet	8 words — < 1%
69	www.cell.com<br>Internet	8 words — < 1%
70	www.ijbs.com<br>Internet	8 words — < 1%
71	www.science.gov<br>Internet	8 words — < 1%
72	Dimitrios Giannis, Dimitrios Moris, Andrew S. Barbas. "Diagnostic, Predictive and Prognostic Molecular Biomarkers in Pancreatic Cancer: An Overview for Clinicians", Cancers, 2021<br>Crossref	7 words — < 1%
73	Elif Değerli, Vildan Torun, Demet Cansaran-Duman. "miR-185-5p response to usnic acid suppresses proliferation and regulating apoptosis in breast cancer cell by targeting Bcl2", Biological Research, 2020<br>Crossref	7 words — < 1%
74	Guangchuang Yu, Li-Gen Wang, Yanyan Han, Qing-Yu He. "clusterProfiler: an R Package for Comparing Biological Themes Among Gene Clusters", OMICS: A Journal of Integrative Biology, 2012<br>Crossref	7 words — < 1%
75	Kun Cai, Shiyu Chen, Changhao Zhu, Lin Li, Chao Yu, Zhiwei He, Chengyi Sun. "FOXD1 facilitates pancreatic cancer cell proliferation, invasion, and metastasis by regulating GLUT1-mediated aerobic glycolysis", Cell Death & Disease, 2022<br>Crossref	7 words — < 1%

76 Cheng-Cao Sun, Ling Zhang, Guang Li, Shu-Jun Li et al. "The lncRNA PDIA3P Interacts with miR-185-5p to Modulate Oral Squamous Cell Carcinoma Progression by Targeting Cyclin D2", Molecular Therapy - Nucleic Acids, 2017 — 6 words — < 1%

Crossref

---

77 Divya Murthy, Kuldeep S. Attri, Pankaj K. Singh. "Phosphoinositide 3-Kinase Signaling Pathway in Pancreatic Ductal Adenocarcinoma Progression, Pathogenesis, and Therapeutics", Frontiers in Physiology, 2018 — 6 words — < 1%

Crossref

---

78 Gay, A.N.. "Granulocyte colony stimulating factor alters the phenotype of neuroblastoma cells: implications for disease-free survival of high-risk patients", Journal of Pediatric Surgery, 200805 — 6 words — < 1%

Crossref

---

79 Jigang Bai, Bowen Yao, Liang Wang, Liankang Sun, Tianxiang Chen, Runkun Liu, Guozhi Yin, Qiuran Xu, Wei Yang. "lncRNA A1BG-AS1 suppresses proliferation and invasion of hepatocellular carcinoma cells by targeting miR-216a-5p", Journal of Cellular Biochemistry, 2018 — 6 words — < 1%

Crossref

---

80 Maria Magdalena Barreca, Chiara Zichittella, Riccardo Alessandro, Alice Conigliaro. "Hypoxia-Induced Non-Coding RNAs Controlling Cell Viability in Cancer", International Journal of Molecular Sciences, 2021 — 6 words — < 1%

Crossref

---

81 Sirkeci, Esra Yıldırım. "Network-Based Integration of Multi-Omic Profiles to Purpose New Biomarker Candidates in Pancreatic Cancer", Marmara Universitesi (Turkey), 2023 — 6 words — < 1%

ProQuest

82 Wei Ni, Yaoxiong Xia, Yuxu Bi, Fan Wen, Dong Hu, Lin Luo. "FoxD2-AS1 promotes glioma progression by regulating miR-185-5P/HMGA2 axis and PI3K/AKT signaling pathway", Aging, 2019
Crossref
6 words — < 1%

83 Xueke Wang, Meisong Kang, Chun Liu, Ting Lin, Xiao Han, Xiwen Jiang. "Current State and Progress of Research on the Role of lncRNA in HBV-Related Liver Cancer", Frontiers in Cellular and Infection Microbiology, 2021
Crossref
6 words — < 1%

84 Yi Zhang, Yuzhi Wang, Jianlin Chen, Yu Xia, Yi Huang. "A programmed cell death-related model based on machine learning for predicting prognosis and immunotherapy responses in patients with lung adenocarcinoma", Frontiers in Immunology, 2023
Crossref
6 words — < 1%

85 Yibin Deng, Zhongheng Wei, Meijin Huang, Guidan Xu, Wujun Wei, Bin Peng, Shunqiang Nong, Houji Qin. " Long non-coding RNA F11-AS1 inhibits HBV-related hepatocellular carcinoma progression by regulating NR1I3 binding to microRNA-211-5p ", Journal of Cellular and Molecular Medicine, 2019
Crossref
6 words — < 1%

EXCLUDE QUOTES	OFF	EXCLUDE SOURCES	OFF
EXCLUDE BIBLIOGRAPHY	ON	EXCLUDE MATCHES	OFF
